# Exploring the “gene–protein–metabolite” network of coronary heart disease with phlegm and blood stasis syndrome by integrated multi-omics strategy

**DOI:** 10.3389/fphar.2022.1022627

**Published:** 2022-11-29

**Authors:** Guang Yang, Siyuan Zhou, Haoqiang He, Zinuo Shen, Yongmei Liu, Jun Hu, Jie Wang

**Affiliations:** ^1^ Department of Cardiology, Guang’anmen Hospital, China Academy of Chinese Medical Sciences, Beijing, China; ^2^ School of traditional chinese medicine, Beijing University of Chinese Medicine, Beijing, China

**Keywords:** coronary heart disease, phlegm and blood stasis syndrome, traditional Chinese medicine, RNA-seq, proteomics, metabolomics

## Abstract

**Background:** According to the theory of traditional Chinese medicine, phlegm and blood stasis (PBS) is the pathological basis for coronary heart disease (CHD). This study aimed to explore the biological basis of PBS syndrome in CHD.

**Methods:** Using a strategy that integrated RNA-seq, DIA-based proteomics, and untargeted metabolomics on 90 clinic samples, we constructed a “gene–protein–metabolite” network for CHD-PBS syndrome. We expanded the sample size and validated the differential genes and metabolites in the network through enzyme-linked immunosorbent assay.

**Results:** Our findings revealed that the “gene–protein–metabolite” network of CHD-PBS syndrome included 33 mRNAs, four proteins, and 25 metabolites. JNK1, FOS, CCL2, CXCL8, PTGS2, and CSF1 were all poorly expressed in the PBS group during the sequencing stage, whereas arachidonic acid (AA) was highly expressed. During the validation stage, JNK1, AP-1, CCL2, and CXCL8 were poorly expressed, whereas PTGS2, CSF1, and AA were highly expressed. The area under the receiver operating curve was as follows: CSF1 [0.9635, 95%CI (0.9295, 0.9976)] >JNK1 [0.9361, 95% CI (0.8749, 0.9972)] >CXCL8 [0.8953, 95% CI (0.8222, 0.9684)] > CCL2 [0.8458, 95% CI (0.7676, 0.9241)] >AP-1 [0.7884, 95%CI (0.6869, 0.8899)]. The logistic regression model composed of CSF1 and JNK1 showed the greatest diagnostic value and significance for PBS syndrome.

**Conclusion:** PBS syndrome is characterized by low levels of FOS, AP-1, CCL2, CXCL8, and JNK1 and elevated levels of PTGS2 and CSF1, implying that the AA metabolism is abnormal and that the JNK/AP-1 pathway is inhibited. PBS syndromes, as a subtype of CHD, may have unique molecular changes. Background. Globally, coronary heart disease (CHD) is the leading cause of death, and this would likely continue until 2030 (Mirzaei et al., 2009, 95, 740–746). According to the disease course, CHD can be classified as chronic stable CHD (or chronic coronary syndrome) and acute coronary syndrome (ACS) (Katus et al., 2017; Knuuti, 2019). Although stable CHD is not as lethal as ACS, it has a varied incidence range and patients with CHD have prolonged angina. Some symptoms of stable angina are alleviated with pharmacological therapy, but it cannot eliminate recurrent angina (Rousan et al., 2017). The clinical outcomes were not significantly improved in patients who underwent revascularization compared with those who received optimal pharmacological therapy (Shaw et al., 2008; Antman and Braunwald, 2020). A bottleneck appears to exist in CHD treatment, and traditional Chinese medicine (TCM) can act as a favorable complement. Because of its individualized treatment approach, TCM is widely practiced in eastern civilizations (Teng et al., 2016). TCM has become a principal complement in western countries (Wieland et al., 2013). Like “disease” is used in western medicine, “syndrome” is used in TCM to comprehend anomalous human conditions on the basis of patients’ symptoms, tongue, and pulse (Li et al., 2012). On the basis of disease-syndrome diagnose, a TCM doctor can subclassify CHD patients into various categories, such as phlegm and blood stasis (PBS) syndrome, cold congealing and Qi stagnation syndrome, and Qi stagnation and blood stasis syndrome. PBS syndrome has recently emerged as a hot research topic in the TCM field. Objective diagnosis, expert consultations, and efficacy evaluation scales have been developed for PBS syndrome (Ren et al., 2020; Liu et al., 2021; Zheng et al., 2022). The concept of “omics” originates from the genome. It refers to the vocabulary generated by biological molecules at different levels to describe high-sequence molecular biological data resources (Dai and Shen, 2022). RNA, protein, and metabolites decipher the essence of complex etiologies, and the integration of transcriptomics, proteomics, and metabolomics are becoming a promising research mode (Pan et al., 2022). Multi-omics studies have revealed the biological characteristics of APOE transgenic mice, bronchopulmonary dysplasia, and plant tolerant to heavy metals (Singh et al., 2016; Lal et al., 2018; Mohler et al., 2020). Over the past few years, many academic achievements related to CHD-PBS syndrome have been accrued in the single-omic area. For example, Zhou identified the differential metabolites between PBS syndrome and Qi and Yin deficiency syndrome by using the urine samples of 1072 volunteers. Some of the specific metabolites of PBS syndrome are pyroglutamic acid, glutaric acid, glucose, mannitol, and xanthine (Zhou et al., 2019). Li’s metabolomic study suggested that valine, leucine, isoleucine, and glycerol phospholipid metabolism could represent PBS syndrome (Zheng et al., 2022). Although some progress has been made in the understanding of PBS syndrome in CHD through the studies conducted, some issues still exist, such as a single-omics level, a lack of in-depth research, an inability to verify each other’s research results, and a lack of validation of research conclusions. Overall, a systematic description of the biological foundation of PBS syndrome is lacking. Thus, the present study utilizes system biology methodologies and constructs a multi-omics network by integrating differential genes, proteins, and metabolites to systematically and comprehensively reveal the biological basis of CHD-PBS syndrome. The current study explored 1) the characteristics of the transcriptome, proteome, and metabolome for CHD-PBS syndrome; 2) the “gene–protein–metabolite” network based on differential genes (DGs), differential proteins (DPs), and differential metabolites (DMs); 3) the key biological process and metabolic pathway most related to PBS syndrome; and 4) quantitative results and the diagnostic potential of biomarkers for PSB syndrome. Materials and methods. Multi-omics sequencing, bioinformatics analysis, and clinical validation research strategy. We collected the blood samples from healthy subjects as well as CHD patients with PBS and non-phlegm and blood stasis (NPBS) syndrome to compare the differences between them by subjecting the samples to the transcriptome, proteome, and metabolomics analyses. Bioinformatics analysis identified differential molecules as well as related biological processes and pathways. Next, the “gene–protein–metabolite” network was constructed using the MetaboAnalyst database, String database, and Cytoscape software. We selected molecules with strong centrality and biological association as potential PBS syndrome biomarkers and recruited more volunteers for further validation by enzyme-linked immunosorbent assay (ELISA). Finally, the ROC curve was utilized to assess the level and diagnostic efficacy of various molecules (Figure 1).

## Participants recruitment

The RNA-seq-based transcriptomic analysis included 15 participants, five each with CHD-PBS syndrome and CHD-NPBS syndrome and five healthy controls (HC). The data-independent-acquisition (DIA) proteomic study included 18 participants, six each with CHD-PBS syndrome and CHD-NPBS syndrome and six HC. The metabolomic study included 30 patients each with CHD-PBS syndrome and CHD-NPBS syndrome and 30 HC. The diagnostic test included 94 participants, of which 64 patients had CHD-PBS syndrome and 30 were HC. The informed consent forms were signed voluntarily by all participants. The CHD patients were recruited from cardiovascular ward of Guang’anmen Hospital and outpatient clinic between August 2020 to March 2021. The healthy adults were enrolled when they showed normal physical examination. One Chief Physician of Cardiovascular Department in Guang’anmen Hospital (J Hu) determined whether patients met the inclusion criteria and Exclusion criteria, with verification by three assistants (G Yang, HQ He, and SY Zhou). The ethics committee of Guang 'anmen Hospital approved the study (No. 2019-225-KY).

### Inclusion criteria

The inclusion criteria were as follows: patients diagnosed with CHD-PBS syndrome and CHD-NPBS syndrome; age 30–75 years; voluntary participation. The disease diagnosis was made with reference to the 2019 European Society of Cardiology Guidelines for the Diagnosis and Management of Chronic Coronary Syndromes (Knuuti, 2019). The syndrome diagnosis was referred to the Diagnostic Criteria for Syndrome Elements of Coronary Heart Disease Angina, as published by the Chinese Society of Traditional Chinese Medicine Cardiovascular Disease Branch in 2018 (Wang and Xing, 2018) (Supplementary Figure S1). One syndrome was diagnosed when the total symptom score was >8. PBS syndrome was diagnosed when both phlegm syndrome and blood stasis syndrome criteria were met. Otherwise, the condition was diagnosed as NPBS syndrome.

**FIGURE 1:**
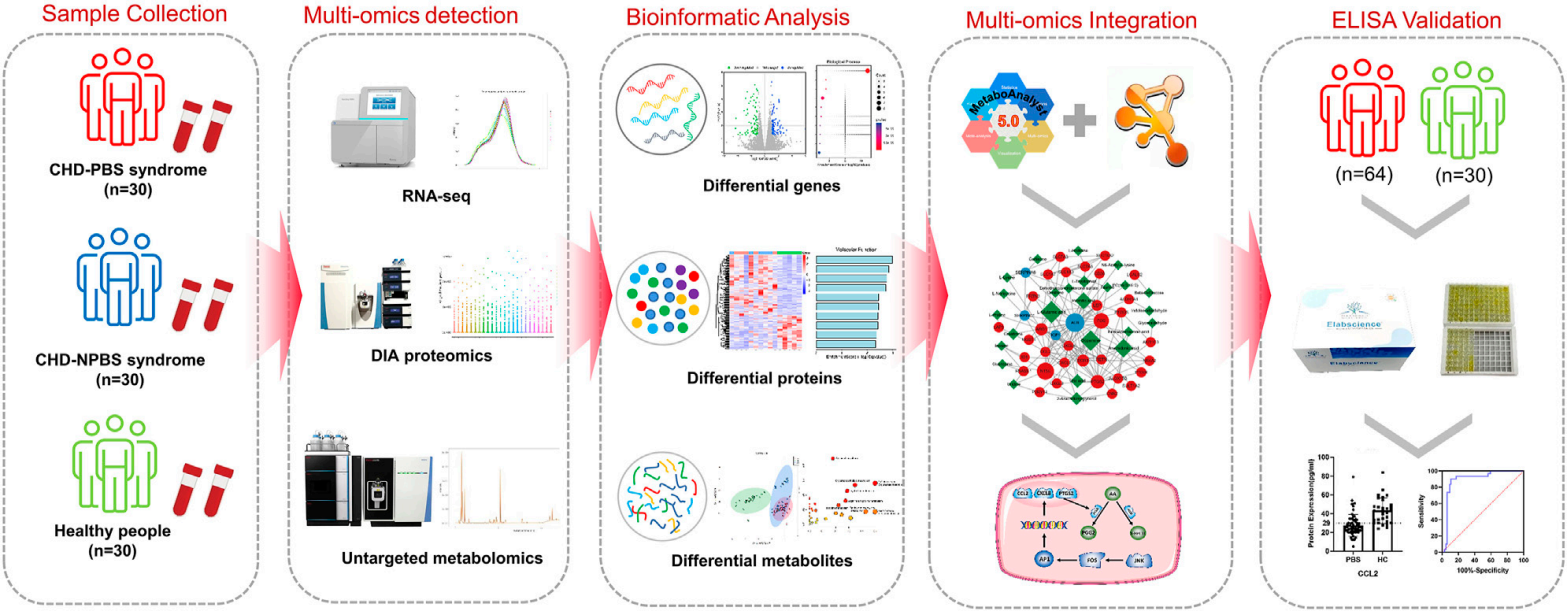
The flowchart of the integrated multi-omics strategy for PBS syndrome.

### Exclusion criteria

Participants meeting any of the following criteria would be excluded from the study. 1) Patients with a history of unstable angina or acute myocardial infarction; other conditions that can cause chest pain, including congenital heart disease, valvular disease, and severe neurological disorders; arrhythmias; 2) Patients with the acute phase of cerebral infarction. 3) Patients with severe liver and kidney diseases; patients with alanine aminotransferase and aspartate aminotransferase 1.5 times higher than the normal upper limit; patients with abnormal renal function. 4) Infection acute within the past 2 weeks. 5) Patients with other life-threatening conditions requiring treatment (e.g., t hematological diseases, umors, diabetic complications, etc.)

### RNA-seq-based transcriptomic study

#### Sample collection and RNA extraction

Fasting venous blood samples were collected from the subjects in the early morning and dispensed into 2 ml EDTA-containing anticoagulation tubes. The tubes were centrifuged for 15 min and the supernatant was discarded. The precipitate was then transferred into a 15-ml centrifuge tube, to which an erythrocyte lysate was added in a ratio of 1:3. The tube was allowed to sit for 10 min and centrifuged again. After the centrifugation, the supernatant was discarded and the erythrocyte lysate was added at a ratio of 1:1 and centrifuged again. After centrifugation, the supernatant was discarded and the precipitate was transferred into a 2-ml lyophilization tube. To this tube, 1.5 ml of Trizol was added and the tube was placed in a −80°C refrigerator for freezing. Total RNA was extracted using the mirVana miRNA Isolation Kit (Ambion) as per the manufacturer’s protocol. RNA purity and quantification were evaluated using the NanoDrop 2000 spectrophotometer. RNA integrity was assessed using the Agilent 2100 Bioanalyzer.

#### Library construction and sequencing

The RNA library was completed by Shanghai OE Biotech Co., Ltd. The libraries were constructed using the TruSeq Stranded mRNA LT Sample Prep Kit (Illumina, San Diego, CA, United States) according to the manufacturer’s instructions. These libraries were then sequenced on the Illumina HiSeq X Ten platform to generate 150-bp paired-end reads. About 49 M raw reads for each sample were generated in all. Raw data (raw reads) in the fastq format were first processed using Trimmomatic and the low-quality reads were removed to obtain clean reads. Then, about 48.8 M clean reads for each sample were retained for subsequent analyses. The clean reads were mapped to the human genome (GRCh38) using HISAT2. FPKM of each gene was calculated using Cufflinks, and the read counts of each gene were obtained by HTSeqcount.

#### Differential gene expression analysis

Differential gene expression analysis was performed using the DESeq2 package for the R software. *p* < 0.05 and foldchange(FC) > 2 or foldchange <0.5 was set as the threshold for DGs. Hierarchical cluster analysis of DGs was performed to demonstrate the expression pattern of genes in different groups and samples. GO enrichment and KEGG pathway enrichment analysis of DGs were respectively performed using the R software based on the hypergeometric distribution.

## Data-independent-acquisition-based proteomic study

### Plasma protein extraction

Fasting venous blood samples of the participants were collected in the morning and subpacked into a 2-ml EDTA-containing anticoagulation tube for centrifugation. The supernatant was centrifuged at 3000 ×*g* at 4°C for 15 min and the fresh supernatant was collected into an Eppendorf tube. Proteins were extracted and identified by Shanghai OE Biotech Co., Ltd, followed by their labeling and storage in a refrigerator at −80°C. The total protein content in the sample was determined; half of the protein was subjected to protein concentration determination and SDS-PAGE detection, and the remaining half was subjected to trypsin enzymolysis. After desalting the enzymolysis peptide, the samples were identified by liquid chromatography-tandem mass spectrometry (LC-MS/MS).

### Liquid chromatography-tandem mass spectrometry detection

All analyses were performed by using the Q-Exactive HF mass spectrometer (Thermo, United States) equipped with a Nanospray Flex source (Thermo). Before mass spectrometry detection, each sample was mixed according to the volume ratio iRT: sample to be tested = 1:10 as an internal standard. First, high-pH liquid-phase separation was performed. All samples after enzymatic hydrolysis were mixed with equal amounts of peptides. The Agilent 1100 HPLC system was used to separate the components in the mobile phase with pH = 10. A total of 10 components were collected and vacuum-freeze-dried until further analyses by mass spectrometry. The liquid in the storage ring was pushed into the analysis column connected with the mass spectrometer by a high-pressure pump at the flow rate of 1 μL/min and separated by the 30SPD method. The MS parameters of data-dependent acquisition (DDA) were set as follows: Capillary: 1.5 kV; Dry Temperature: 180°C; Dry Gas: 3.0 L/min. Mass Range: 100–1700 m/z; Ion Mobility: 0.6–1.6; Collision Energy: 20–59 EV. DIA mass spectrum scanning parameters were kept the same as for DDA.

### Liquid chromatography-tandem mass spectrometry data analysis

The MS output was matched with the theoretical spectrogram generated by the fastA library. The original LC-MS/MS file was imported to Spectronaut Pulsar for library search and construction. The main parameters included Missed cleavage: two; Fixed modification: Carbamidomethyl (C); Variable modification: Oxidation (M); Enzyme: Trypsin/P; Protein FDR Cut Off: 0.01; Peptide FDR Cut Off: 0.01; PSM FDR Cut Off: 0.01; Database: Uniprot-REVIEWED -Homo sapiens (Human) -2020081. DIA raw data were processed using the Spectronaut Pulsar software. The key parameters were as follows: Precursor Qvalue cutoff: 0.01; Protein Qvalue cutoff: 0.01; Normalization Strategy: Local Normalization; Quantity MS-Level: MS2.

### Trusted protein analysis

The original data were retrieved from the database, and proteins whose expression value accounted for ≥50% were retained in any group of samples. Proteins with missing values ≤ 50% were filled with the mean value of the same group. The trusted proteins were obtained by Median Normalization and log2 conversion. Log2FC was obtained by subtracting the average value of the trusted proteins in different groups. The fold change was calculated from log2FC, and MS Excel was used to calculate the FC and *q*-value. The screening criteria were FC > 1.2 and *q*-value < 0.05, and the volcano map was drawn with the ggplot2 package of R software for visualization.

### Untargeted metabolomic study

#### Sample preparation

Human fasting venous blood samples were collected from 08:00 AM to 10:00 AM, and the serum was obtained after centrifugation at 3000 ×*g* at 4°C for 10 min. The samples stored at −80°C were thawed at room temperature. Sample preparation and LC-MS detection were completed by Shanghai OE Biotech Co., Ltd. Briefly, 100 μL of the sample was added to a 1.5-ml Eppendorf tube containing 10 μl of 2-chloro-l-phenylalanine (0.3 mg/ml) dissolved in methanol as an internal standard, and the tube was vortexed for 10 s. Subsequently, 300 μl of an ice-cold mixture of methanol and acetonitrile (2/1, v/v) was added to the tube, and the mixture was vortexed for 1 min, ultrasonicated at ambient temperature (25°C–28°C) for 10 min, and stored at −20°C for 30 min. The extract was centrifuged at 13000 rpm, 4°C for 15 min. Then, 300 ml of the supernatant was collected in a brown glass vial and dried in a freeze-concentration centrifugal dryer. Then, 400 μl of a methanol and water mixture (1/4, vol/vol) was added to each sample, vortexed for 30 s, and placed at 4°C for 2 min. The samples were centrifuged at 13000 rpm/4°C for 5 min. The supernatants (150 μl) from each tube were collected using crystal syringes, filtered through 0.22-μm microfilters, and transferred into LC vials. These vials were stored at −80°C until LC/MS analyses.

#### Analysis conditions of LC/MS

The LC-MS system used was the Dionex U3000 UHPLC Ultra-High Performance Liquid and QE PLUS High-resolution Mass Spectrometer under the following chromatographic conditions: chromatographic column: ACQUITY UPLC HSS T3 (100 × 2.1 mm, 1.8 um); column temperature: 45°C; Mobile phase: A-water (containing 0.1% formic acid), B- acetonitrile (containing 0.1% formic acid); Flow rate: 0.35 ml/min; Injection volume: 2 μl. The MS conditions were as follows: Ion source: ESI; Positive and negative ion scanning mode was used to collect the MS signals. Mass spectrum parameters: Spray Voltage (V): 3800 (positive ion), -3000 (negative ion); MS parameters: Spray Voltage (V): 3800 (positive ion), -3000 (negative ion); Capillary Temperature (°C): 320; Aux gas heater temperature (°C): 350; Sheath Gas Flow Rate (Arb): 35; Aux gas flow rate (Arb): 8; S-lens RF level: 50; Mass range (m/z): 100–1000; Full ms resolution: 70,000; MS/MS resolution: 17500; NCE/stepped NCE: 10, 20, 40.

#### Data preprocessing and statistical analysis

The acquired LC-MS raw data were analyzed by the progqenesis QI software (Waters Corporation, Milford, United States) using the following parameters: precursor tolerance set to 5 ppm, fragment tolerance set to 10 ppm, and retention time (RT) tolerance set to 0.02 min. Internal standard detection parameters were deselected for peak RT alignment, and the isotopic peaks were excluded for analysis, with the noise elimination level set at 10.00 and minimum intensity at 15% of the base peak intensity. The Excel file was obtained with 3-dimensional datasets, including m/z, peak RT, and peak intensities, and RT–m/z pairs were used as the identifier for each ion. The resulting matrix was further reduced by removing any peaks with a missing value (ion intensity = 0) in more than 50% of samples. The metabolites were identified by progenesis QI (Waters Corporation, Milford, United States) Data Processing Software, based on public databases and self-built databases. The positive and negative data were combined to obtain the combined data. Orthogonal partial least-squares-discriminant analysis (OPLS-DA) was conducted by the SIMCA software to visualize the metabolic alterations among the experimental groups. The differential metabolites were selected on the basis of a statistically significant threshold of variable influence on the projection (VIP) values obtained from the OPLS-DA model, where metabolites with VIP >1.0 and adj.*p* < 0.05 were considered DMs.

#### Multi-omics network construction and potential biomarkers selection

In order to investigate the biological links between the transcriptome/proteome and metabolome, we entered the DGs, DPs, and DMs into the “Network Analysis” module of the MetaboAnalyst database. The interactions between DGs and DPs were determined by using the String database. We integrated the relevant data in MS Excel and determined the genes, proteins, and metabolites that formed the network. The “gene–protein–metabolite” network of syndromes was further visualized by using the Cytoscape software. We selected genes and metabolites with high centrality and biological linkages as potential biomarkers for PBS syndrome, based on the literature and KEGG enrichment results.

#### ELISA validation

We enlarged the sample size by collecting serum from 64 CHD-PBS syndrome patients and 30 HC. To conduct the validation experiment, we purchased ELISA kits from Elabscience (China) and followed the manufacturer’s instructions. First, we added 50 μl of a diluted standard and sample to a 96-well plate, immediately followed by the addition of 50 μl of the Biotinylated Detection Ab working solution to each well. The plate was covered with a sealer and incubated for 45 min at 37°C. The solution was then decanted and 350 μl of a wash buffer was added to each well. After repeating the washing step thrice, 100 μL of the HRP conjugate working solution was added to each well. The plate was covered with a new sealer and incubated for another 30 min at 37°C. The solution was then decanted from each well and the washing process was repeated 5 times. Then, 90 μl of the substrate reagent was added to each well. The plate was covered with a new sealer and incubated for about 15 min at 37°C. Finally, 50 μL of the stop solution was added to each well and the optical density (OD value) of each well was determined with the microplate reader (Tecan, Switzerland) at 450 nm.

## Results

### Clinical characteristics of coronary heart disease patients with phlegm and blood stasis or non-phlegm and blood stasis syndrome

**FIGURE 2:**
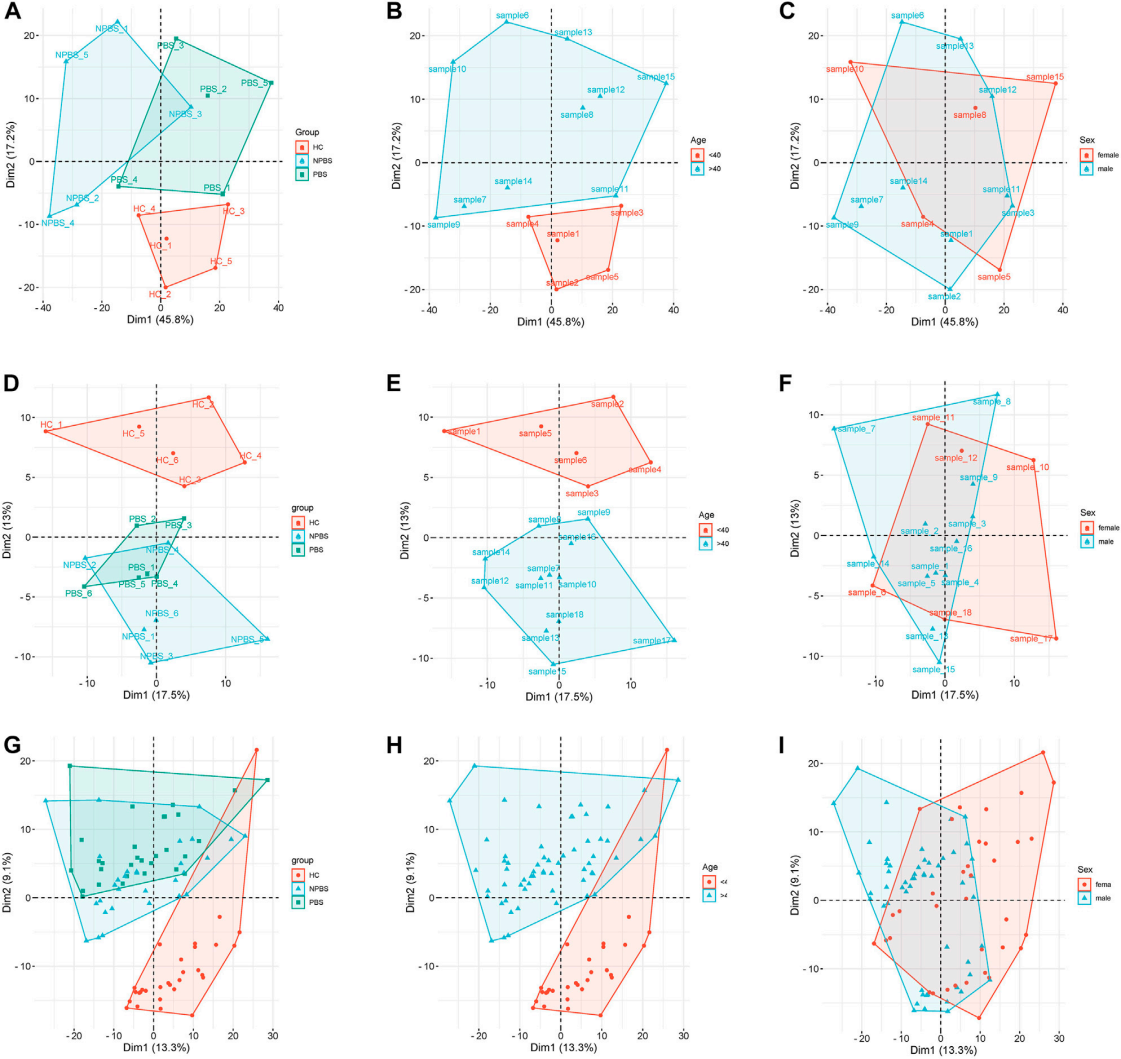
Principal component analysis for different clinical characteristics. **(A–C)** The influence of disease, age, and sex on transcriptome expression. **(D–F)** The influence of disease, age, and sex on proteome expression. **(G–I)** The influence of disease, age, and sex on metabolome expression.

Table 1 lists the demographic and clinical characteristics of participants. No significant differences in age, sex, and past medical history were observed between the CHD-PBS and CHD-NPBS groups. However, disparities in age and past medical history were observed between CHD patients and healthy adults (Table 1). We evaluated the influence of disease, age, and sex on transcriptome and proteome, and metabolome expression separately. Principal component analysis was performed using the selected top 1000 genes and metabolites, and all proteins. According to the results, disease status and age were the main factors resulting in changes in the transcriptome, proteome, and metabolome (Figures 2A–F). Obviously, significant differences are present in the expression of mRNA, proteins, and metabolites between patients with CHD-PBS syndrome, patients with CHD-NPBS syndrome, and healthy adults. In the following study, we will quantitatively describe this difference and determine the association between the different omics.

**TABLE 1:** Clinical characteristics of CHD patients with either PBS or NPBS syndrome.

Participants	CHD-PBS	CHD-NPBS	HC
RNA-seq			
sample size	5	5	5
Age	60.20 ± 13.63	63.20 ± 3.49	25.00 ± 1.87
Sex (male%)	4 (80.00%)	3 (60.00%)	3 (60.00%)
Hypertension (%)	4 (80.00%)	3 (60.00%)	0
Hyperlipidemia (%)	2 (40.00%)	5 (100.00%)	0
Diabetes (%)	3 (60.00%)	1 (20.00%)	0
DIA-based proteomics			
sample size	6	6	6
Age	59.33 ± 12.37	63.17 ± 3.13	25.00 ± 1.67
Sex (male%)	5 (83.33%)	4 (66.67%)	3 (50.00%)
Hypertension (%)	4 (66.67%)	3 (50.00%)	0
Hyperlipidemia (%)	3 (50.00%)	5 (83.33%)	0
Diabetes (%)	3 (50.00%)	2 (33.33%)	0
Metabolomics			
sample size	30	30	30
Age	60.04 ± 7.40	65.37 ± 3.91	25.03 ± 1.92
Sex (male%)	23 (76.67%)	15 (50.00%)	16 (53.33%)
Hypertension (%)	27 (90.00%)	20 (66.67%)	0
Hyperlipidemia (%)	22 (73.33%)	27 (90.00%)	0
Diabetes (%)	12 (40.00%)	11 (36.67%)	0
ELISA validation			
sample size	64	-	30
Age	60.99 ± 7.10	-	57.24 ± 5.51
Sex (male%)	41 (64.06%)	-	17 (53.33%)
Hypertension (%)	39 (60.94%)	-	0
Hyperlipidemia (%)	51 (79.69%)	-	0
Diabetes (%)	18 (28.13%)	-	0

### Transcriptomic characteristics of CHD-PBS syndrome

**FIGURE 3:**
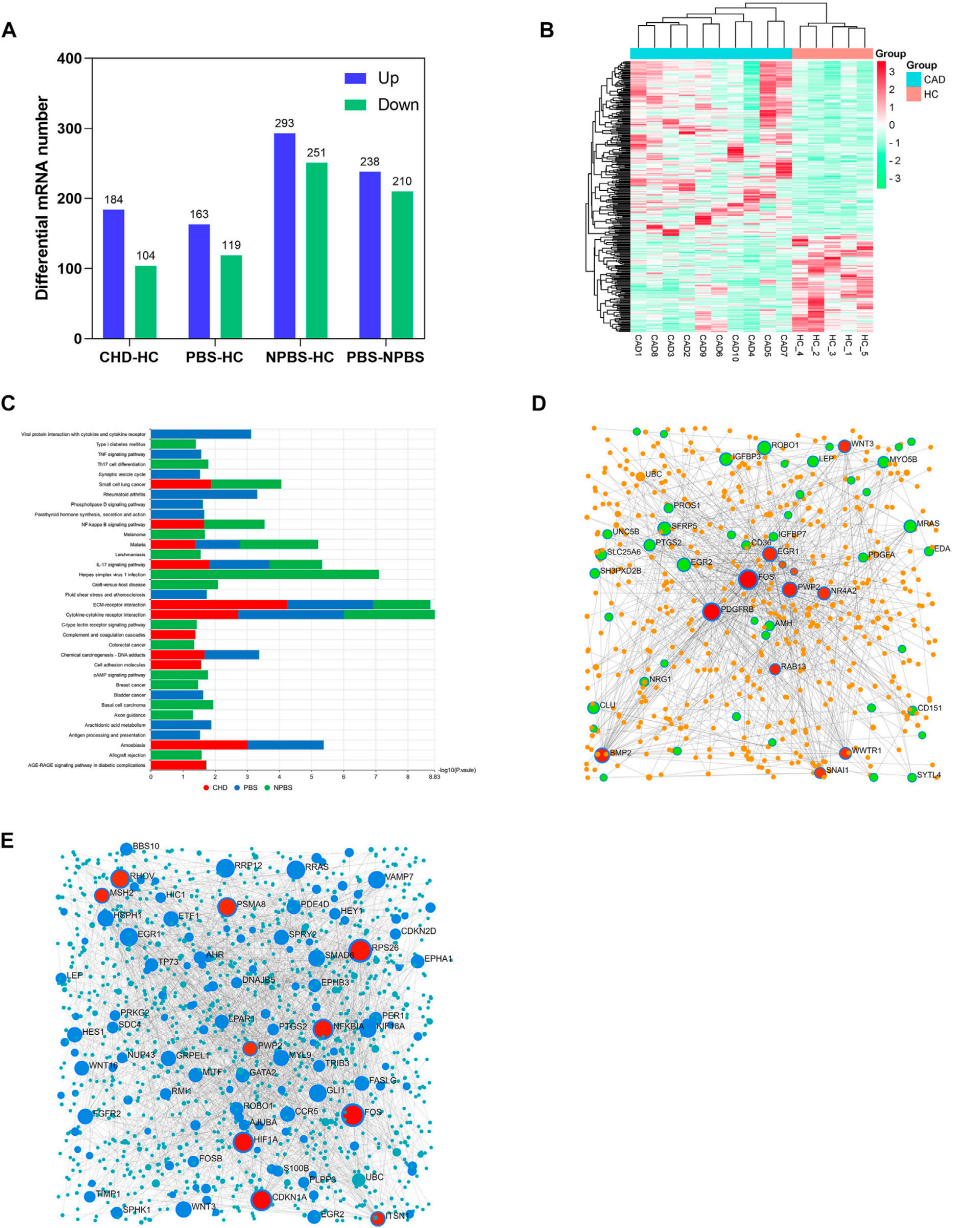
Transcriptomics characteristic of CHD-PBS syndrome. **(A)** The number of differentially expressed genes in each group. **(B)** Heat map of differential genes between CHD and HC groups. **(C)** Comparison of KEGG pathway in different groups. **(D)** Gene-gene interaction network for CHD-PBS syndrome. **(E)** Gene-gene interaction network for CHD-NPBS syndrome.

In a transcriptomics study, we identified the DGs of CHD-PBS syndrome. First, we assessed the quality of sequencing data. According to the quality assessment of raw data, 93%–96% of bases fulfilled the Q30 requirements for each sample, and the error rate of bases was <0.04%, which indicated that the sequence was stable (Supplementary Table S6). Supplementary Table S7 presents the results of the reference genome comparison. FPKMs can indirectly reflect gene expression. In total, over 20,000 functional genes were detected. The mean value of FPKMs was 15–18, and the FPKM homogeneity was good across individual samples, suggesting that the quality of each sample was good and reliable (Supplementary Table S8; Supplementary Figure S1). To identify the DGs, we performed a count-based DG expression analysis. In total, 288 DGs were identified between the CHD and HC groups, with 184 DGs upregulated and 104 DGs downregulated (Figure 3A). The heatmap revealed significant differences in gene expression between the CHD and HC groups (Figure 3B). Based on diagnostic criteria for syndromes, the samples were divided into two groups, as detailed in Supplementary Table S1. Compared with the HC group, the PBS group had 282 genes; of them, 163 genes were upregulated and 169 genes were downregulated. The NPBS group contained 544 genes, with 293 genes upregulated and 251 genes downregulated. In addition, 448 DGs were identified between the PBS and NPBS groups. Then, KEGG enrichment analysis was performed for DGs of CHD (*q*-value < 0.05). The results revealed that these DGs were enriched in the AGE-RAGE signaling pathway, complement and coagulation cascades, IL-17 signaling pathway, and NF-κB signaling pathway (Supplementary Figure S2A). The DGs of PBS syndrome were enriched in atherosclerosis, arachidonic acid (AA) metabolism, parathyroid hormone metabolism, the phospholipase D signal transduction pathway, the TNF signal transduction pathway, bladder cancer, and rheumatoid arthritis. The DGs of NPBS syndrome were enriched in the c-type lectin receptor signal transduction pathway, Th17 cell differentiation, and colorectal cancer (Supplementary Figure S2B,C). On summarizing the enrichment results of the aforementioned three groups, we found that DGs of PBS syndrome were enriched in the TNF signaling pathway, phospholipase D signaling pathway, and AA metabolism (Figure 3C). To further investigate the core genes expressed in patients with the two syndromes, we performed a network analysis of gene–gene interactions. Some of the hub genes for PBS syndrome are FOS, PDGFRB, PWP2, BMP2, and EGR1 (Figure 3D), whereas that for NPBS syndrome were FOS, CDKN1A, HIF, and NFKBIA (Figure 3E).

### Proteomic characteristics of CHD-PBS syndrome

**FIGURE 4:**
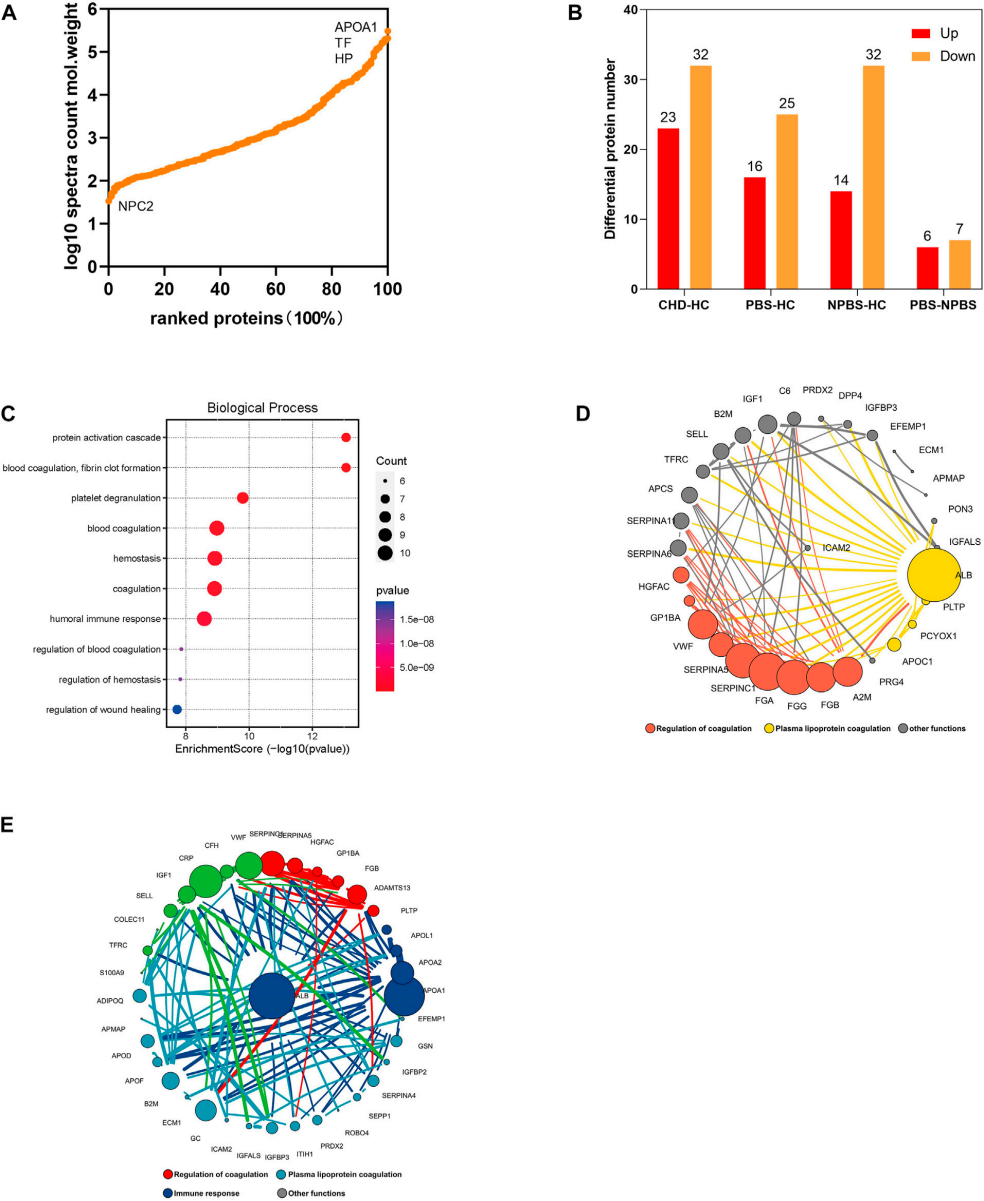
Proteomics characteristic of CHD patients with PBS syndrome. **(A)** Relative abundance of proteins spans five orders of magnitude. **(B)** The number of differential proteins in each group. **(C)** GO enrichment analysis of differential proteins in patients with CHD-PBS Syndrome. **(D)** Protein-protein interactions analysis of differential proteins in patients with CHD-PBS syndrome. **(E)** Protein-protein interactions analysis of differential proteins in patients with CHD-NPBS syndrome.

Next, we performed a DIA-based proteomic analysis to explore the differences in protein abundance among PBS syndrome and healthy adults. The average deviation and DIA data points of each sample satisfied the standards, demonstrating that this proteome quantification was accurate (Supplementary Figure S3, 4). For monitoring the stability of LC/MS data and the reliability of qualitative and quantitative data, QC samples were inserted into a certain number of samples at each interval in the sample queue. A correlation analysis was conducted, and the correlation coefficients were found to be all >0.9 (Supplementary Figure S5). In total, 3,690 peptides and 324 proteins were discovered in the plasma of 18 participants by using the DIA method. The relative abundance of proteins spanned five orders of magnitude. The highest protein expression level was observed for apolipoprotein A-I (APOA1), whereas the lowest was for NPC intracellular cholesterol transporter 2 (Figure 4A). According to the DP expression analysis, 23 plasma proteins were upregulated and 32 were downregulated in the CHD group compared with the HC group. The PBS syndrome group exhibited 41 DPs, and of them, 16 DPs were upregulated and 25 were downregulated. The NPBS syndrome group exhibited 46 DPs, of which 14 DPs were upregulated and 32 were downregulated compared with the HC group. Supplementary Figure S6 presents the volcano diagrams of the DPs. In addition, 13 DPs were identified between the PBS and NPBS groups, and of them, six were upregulated and seven were downregulated (Figure 4B). The plasma proteome of the two syndromes did not differ as much as the transcriptome. GO annotation analysis revealed that DPs for PBS syndrome were enriched in biological processes such as complement activation, platelet degranulation, humoral immune response, phagocytosis, receptor-mediated endocytosis, classical pathway, and immunoglobulin-mediated immune response (Figure 4C). Next, we determined the protein difference between the two syndromes of CHD. According to the PPI network and analysis of the biological function of core proteins, proteins expressed for PBS syndrome were primarily associated with coagulation and lipid metabolism (Figure 4D), whereas those expressed for NPBS syndrome were associated with coagulation, lipid metabolism, and inflammation (Figure 4E).

### Metabolomic characteristics of CHD-PBS syndrome

**FIGURE 5:**
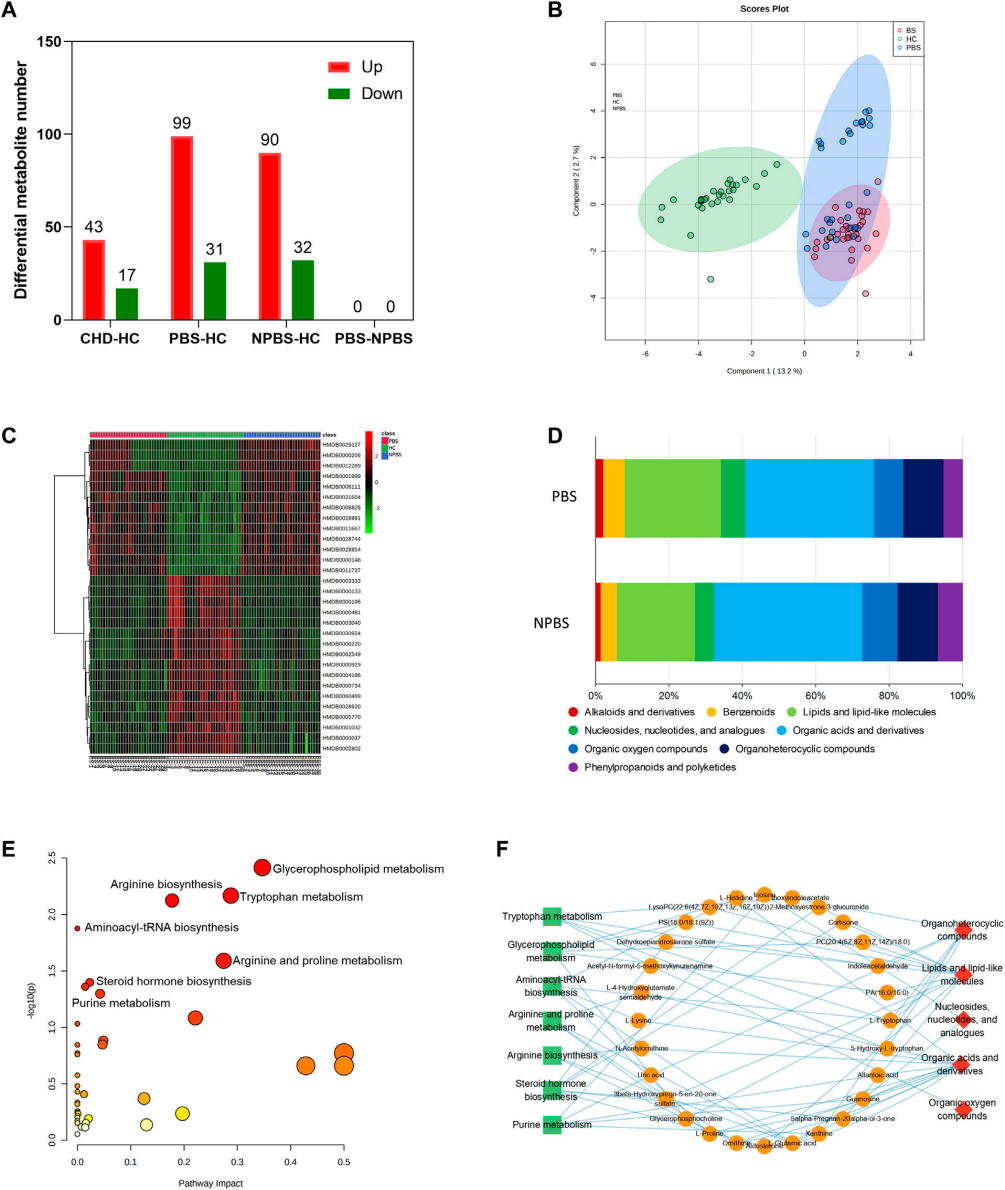
Metabolomics characteristic of CHD patients with PBS Syndrome. **(A)** Number of differential metabolites in each group. **(B)** OPLS-DA analysis of three groups. **(C)** Cluster heatmap analysis of three groups. **(D)** Metabolite type of different syndromes. **(E)** Functional enrichment analysis of identified DMs of PBS syndrome. **(F)** The network relationship of metabolites, species, and enrichment pathways.

Then, we investigated the serum metabolomic characteristics of CHD-PBS syndrome by using positive and negative ion scan data collected from patients and healthy participants through UPLC/MS (Supplementary Figure S7). QC samples were used to further evaluate detection stability. The PCA model diagram derived through 7-fold cross-validation and the Boxplot generated on the basis of the intensity of metabolites demonstrated that the QC samples were closely clustered, which indicated that the detection stability in this study was good (SupplementryFigures S8, 9). In total, 17,890 metabolites were discovered, of which 1385 were annotated in the HDMB database. We generated DMs through SIMCA software’s OPLS-DA analysis, with the screening criteria of VIP >1 and *adj. p-*value < 0.05. Compared with the HC group, 130 (99 upregulated and 31 downregulated DMs) and 122 (90 upregulated and 32 downregulated DMs) metabolites were altered in the CHD-PBS and CHD-NPBS groups, respectively (Figure 5A). The OPLS-DA score and cluster heatmap revealed that the PBS, NPBS, and HC groups exhibited a clear trend of separation, whereas the PBS and NPBS groups somewhat overlapped (Figures 5B,C). On differentiating the two syndromes by the metabolite type, we found that the PBS group exhibited more lipid trends (Figure 5D). Next, we used the MetaboAnalyst platform to perform functional enrichment analysis. Several metabolic pathways related to PBS syndrome were identified (*p*-value < 0.05), including tryptophan metabolism, glycerophospholipid metabolism, aminoacyl-tRNA biosynthesis, arginine and proline metabolism, arginine biosynthesis, steroid hormone biosynthesis, and purine metabolism (Figure 5E). To further comprehend the relationship between the metabolites, their species, and enrichment pathways, we edited the results using Cytoscape Software. Lipids and lipid-like molecules, organic acids and their derivatives, and organoheterocyclic compounds were the main species of these metabolites that are involved in the metabolic imbalance observed in patients with PBS syndrome, as depicted in Figure 5F.

### Integrated analysis of DGs, DPs, and DMs

**FIGURE 6:**
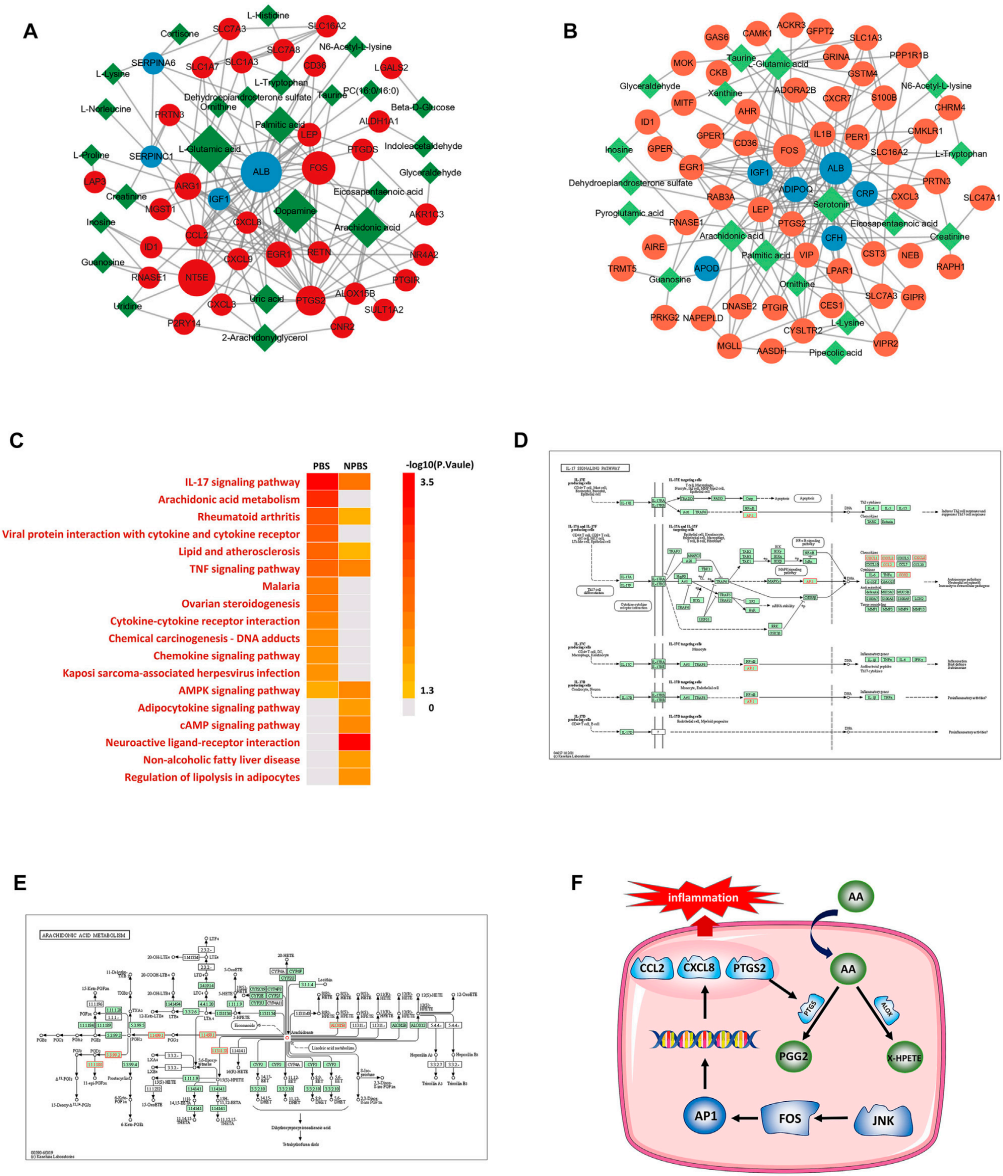
Transcriptome-proteome-metabolome integrated analysis and potential targets. **(A)** Network anlaysis of differential genes, proteins and metabolites for PBS Syndrome. **(B)** Network anlaysis of differential genes, proteins and metabolites for NPBS Syndrome. **(C)** KEGG enrichment analysis for the networks of two Syndromes. **(D)** IL-17 signaling pathway. **(E)** Arachidonic acid metabolism. **(F)** JNK-FOS-AP-1 pathway.

By integrating transcriptomics, proteomics, and metabolomic data, we established a “gene–protein–metabolite” network for the two syndromes. The multi-omics network of PBS syndrome consisted of 33 mRNAs, four proteins, and 25 metabolites (Figure 6A), while that of NPBS syndrome contained 64 mRNAs, nine proteins, and 23 metabolites (Figure 6B). Next, KEGG enrichment analysis performed for the two networks revealed that molecules in the PBS syndrome network were enriched in the IL-17 signaling pathway, rheumatoid arthritis, viral protein interaction with cytokine and cytokine receptor, Chagas disease, TNF signaling pathway, malaria, lipid and atherosclerosis, AA metabolism, and so forth (q-value > 0.05). Molecules in the NPBS syndrome network were only enriched in the neuroactive ligand–receptor interaction (q-value > 0.05) (Figure 6C). Finally, we sought the key targets of PBS syndrome. According to KEGG enrichment results, PBS syndrome was closely related to the IL-17 signaling pathway and AA metabolism. Some of the DGs encode the following key molecules of the MAPK signaling pathway, which was downstream of the IL-17 pathway: FOS (AP-1 in Figure 6E), CCL2, CXCL8, CXCL1, CXCL2, and PTGS2. Among the DGs and DMs, AA, PTGS2, ALOX15B, PTGDS, and AKR1C3 are the substrates and key enzymes of AA metabolism (Figure 6F). Moreover, the molecules in the network were ranked according to their degree and betweenness. We observed that CXCL8, CCL2, FOS, PTGS2, and AA had higher centrality (Supplementary Material). Consequently, owing to the biological association and statistical association, we further validated AA, AP-1, PTGS2, CCL2, and CXCL8 as potential targets of PBS syndrome (Figure 6D).

### Validation by the enzyme-linked immunosorbent assay test

**FIGURE 7:**
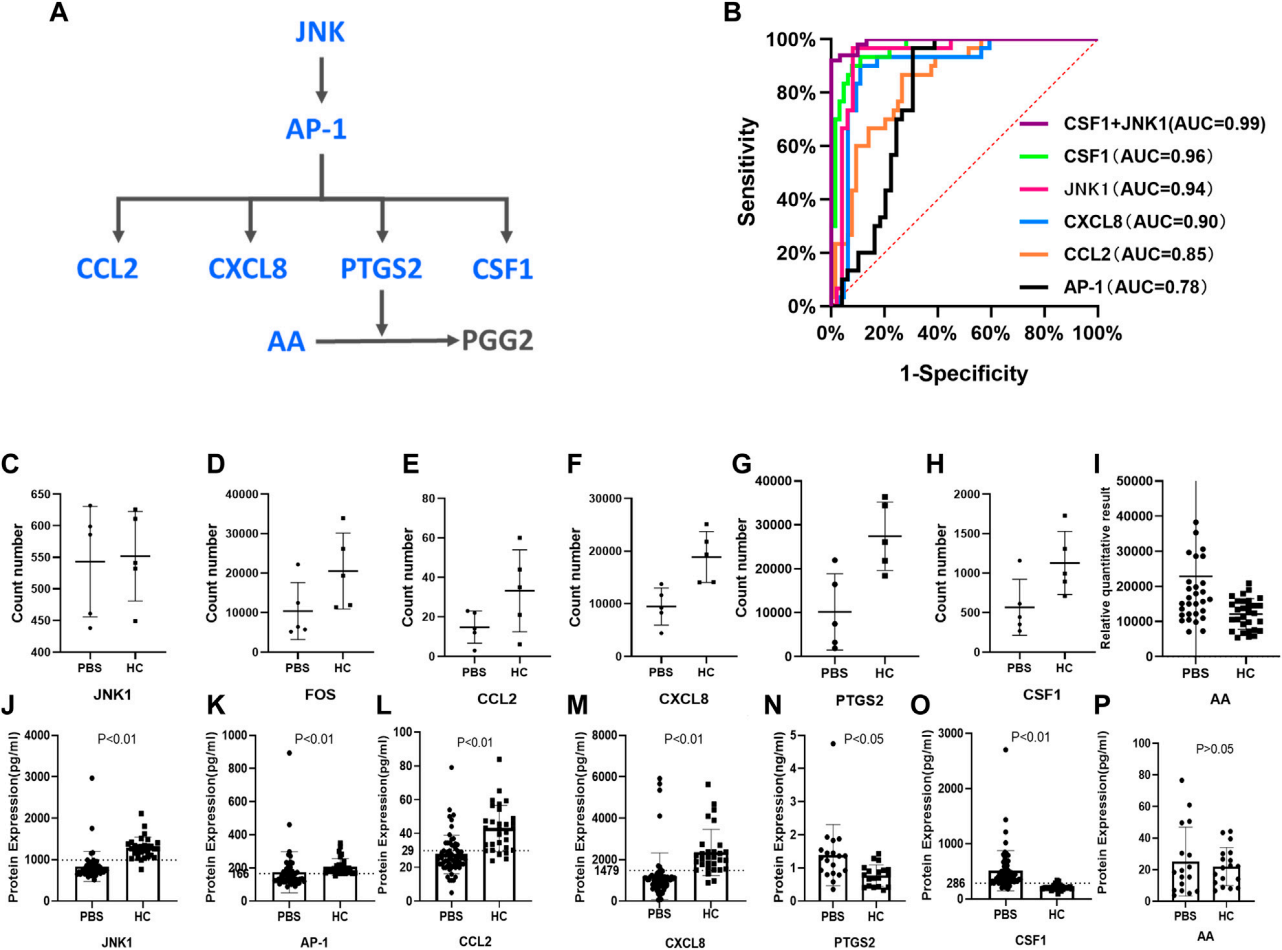
Validation by ELISA test. **(A)** JNK/AP-1 pathway and AA metabolism. **(B)** ROC curves for different molecules. **(C–I)** RNA-seq results of JNK1, FOS, CCL2, CXCL8, PTGS2, CSF1, AA. **(J–P)** ELISA results of JNK1, AP-1, CCL2, CXCL8, PTGS2, CSF1, and AA.

According to the results of the multi-omics network, the hub genes and metabolites for PBS syndrome were AP-1 (FOS), CCL2, CXCL8, PTGS2, and AA. These genes exhibited up–down regulatory relationships and are related to the MAPK pathway (Figure 7A). The FOS family is a subunit of the transcription factor AP-1. Activated JNK phosphorylates FOS and ultimately activates AP-1. Furthermore, CSF1, a downstream molecule of AP-1, was differentially expressed in our transcriptomics study. Based on the aforementioned biological links, AA, JNK1, AP-1, CCL2, PTGS2, CXCL8, and CSF1 were selected as candidate molecules for final validation by expanding the sample size. In the sequencing stage, the expression of JNK1, FOS, CCL2, CXCL8, PTGS2, and CSF1 was low in the PBS group, whereas that of AA was high (Figures 7C–I). In the validation stage, the expression of JNK1, AP-1, CCL2, and CXCL8 was low in the PBS group, whereas that of PTGS2, CSF1, and AA was high (Figure 7J–P). Except for AA, these indicators were all statistically significant (*p* < 0.05). JNK1, AP-1, CCL2, CXCL8, and CSF1 were assessed using the receiver operating characteristic (ROC) curve; these were validated using a large sample size. The bigger the area under the curve (AUC), the more effective the diagnostic value. The AUC of each molecule was as follows: CSF1 [0.9635, 95%CI (0.9295, 0.9976)] > JNK1 [0.9361, 95% CI (0.8749, 0.9972)] > CXCL8[0.8953, 95% CI (0.8222, 0.9684)] > CCL2 [0.8458, 95% CI (0.7676, 0.9241)] > AP-1 [0.7884, 95%CI (0.6869, 0.8899)] (Figure 7B). Logistic regression analysis was conducted based on CSF1 and JNK1, and the regression equation was as follows: *p* = 1/[1+ e^−(0.042×CSF1-0.008×JNK1-2.146)^]. The AUC of this logistic model was 0.992. Detecting CSF1 and JNK1 together was of the highest diagnostic value and significance for PBS syndrome. The Youden index was calculated by combining the specificity and sensitivity of the ROC curve. The cut-off value corresponding to the maximum Youden index was considered the best diagnostic point. The optimal diagnostic points of CSF1, JNK, CXCL8, CCL2, and AP-1 were 286.6, 992.7, 1479, 29.78, and 166.8 pg/ml, respectively.

## Discussion

TCM is personalized medicine that investigates clinical diagnosis and treatment regularities. TCM can serve as a valuable complement to Western medicine’s standardized treatment for CHD and related conditions (Zhou et al., 2014). Currently, multi-omics network research guided by the concept of systems biology, utilizing the most up-to-date high-throughput sequencing and high-resolution MS technology, and utilizing the method of non-targeted screening combined with targeted verification is advantageous in the study of biomarkers and biological foundations of TCM syndromes. For example, Wu explored the biological basis of two CHD syndromes using proteomics, metabolomics, and network pharmacology strategy. The study indicated that downregulated PON1 and ADIPOQ might be potential biomarkers of cold coagulation and blood stasis syndrome and that downregulated APOE and APOA1 might be biomarkers of Qi stagnation and blood stasis syndrome (Wu et al., 2021). Guo integrated untargeted and targeted metabonomics and showed that Shi syndromes involved inflammation and anomalous levels of bioactive phospholipids and antioxidant molecules, while Xu syndromes involved long-chain unsaturated lipid ceramide metabolism and bile acid metabolism (Guo et al., 2021). Using human urine samples, Zhou identified 15 and 12 biomarkers of PBS syndrome and Qi and Yin deficiency syndrome, respectively (Zhou et al., 2019). In this study, we proposed a strategy integrating RNA-seq, proteomics, and metabolomics to explore the biological basis of CHD-PBS syndrome by constructing the “gene–protein–metabolite” network associated with this syndrome.

In the transcriptomics study, 282 mRNAs were identified as DGs for PBS syndrome. They were enriched in the TNF signaling pathway, phospholipase D signaling pathway, and AA metabolism. Forty-one proteins were identified as DPs for PBS syndrome in the proteomics study. They were enriched in the biological processes of complement activation, platelet degranulation, humoral immune response, phagocytosis, receptor-mediated endocytosis, *etc.* A total of 130 metabolites were identified as DMs for PBS syndrome in the metabonomics study. They were enriched in unsaturated fatty acid metabolism (AA metabolism, taurine, and hypotaurine metabolism), amino acid metabolism, and vitamin and sugar acid metabolism. The DGs, DPs, and DMs were integrated to construct a gene–protein–metabolite network. After the biological correlation and order of centrality were integrated, AA, JNK1, FOS, CCL2, CXCL8, PTGS2, and CSF1 were identified as potential biomarkers. ELISA results showed that JNK1, AP-1, CCL2, and CXCL8 levels were significantly lower in CHD-PBS patients than in healthy adults, whereas PTGS2 and CSF1 levels were significantly higher. Based on their AUC values, the combined detection of CSF1 and JNK1 had the greatest diagnostic value for PBS syndrome. The biological foundation of PBS syndrome is speculated to be associated with abnormal AA metabolism and inhibition of the JNK/AP-1 pathway.

### AA metabolism and CHD-PBS syndrome

Previous metabolomics results have shown that glucose and glucose metabolism were significantly increased in patients with CHD-PBS syndrome (Zhou et al., 2019). High glucose levels activate the RAGE-ERK1/2-ICAM-1 pathway, resulting in accelerated atherosclerosis (Pertynska-Marczewska and Merhi, 2015). We here found that PBS syndrome was highly associated with AA metabolism, especially the PTGS2 and LOX-12/15 pathways. AA, the most widely distributed omega-6 polyunsaturated fatty acid in organisms, generates several different bioactive substances through three metabolic pathways: cyclooxygenase (COX/PTGS), lipoxygenase (ALOX12/15), and cytochrome P450 (CYP450) (Hanna and Hafez, 2018). Different enzymes mediate the production of different metabolites having different effects on the heart. For example, CYP450-mediated epoxyeicosatrienoic acids protect the heart, preventing LPS-induced cardiac dysfunction by inhibiting inflammatory molecules released by macrophages (Dai et al., 2015). 12/15-Lipoxygenase produced by ALOX12/15 promotes oxidative stress and myocardial ischemia/reperfusion injury (Zhang et al., 2021). COX-mediated prostaglandins have complex and controversial effects on the heart (Al-Kofahi et al., 2018) and may promote cardiac repair, possibly by interacting with different cells and prostaglandin receptors. Because AA metabolism is diverse and complex, we focused on AA levels and enzyme levels. Although the AA level did not change significantly, the enzyme levels were significantly upregulated in PBS patients. The levels of ALOX12X and 15-HETE increased during the sequencing stage and those of PTGS2 increased during the validation stage. Levels of beneficial AA products decreased, while those of detrimental products increased, leading to the rapid development of both CHD and PBS syndrome.

### JNK/AP-1 signaling pathway and CHD-PBS syndrome

The JNK/AP-1 signaling pathway is supposed to be upregulated in CHD patients, whereas an opposite result was observed in our study, which may be an effect of PBS syndrome. JNK, also known as stress-activated protein kinase (SAPK), regulates the transcription and protein expression of apoptosis- and inflammation-related downstream genes in a transcription-dependent manner. Upon activation with stimulants, JNK moves from the cytoplasm to the nucleus and activates transcription factors such as AP-1 through phosphorylation. AP-1 is composed of FOS and JUN families and is a key transcription factor regulating cell proliferation, differentiation, apoptosis, transformation, migration, and inflammation. After signal regulation, AP-1 mediates the production of PTGS2, CCL2, CXCL8, and other pro-inflammatory cytokines and chemokines (Nakayama et al., 2022; Matsumoto et al., 2007; Du et al., 2020). The FOS family and AP-1 have a crucial role in atherosclerosis and myocardial ischemia. They have different pathogenic effects on vascular smooth muscle cells, endothelial cells, cardiomyocytes, and macrophages. For example, ox-LDL enhances the c-fos transcriptional activity to promote the expression of the low-density lipoprotein receptor, thereby promoting foam cell formation and atherosclerosis (Miao et al., 2022). AP-1 plays the main role in VSMC proliferation, differentiation, and migration and participates in AS formation (Meijer et al., 2012; Zhang et al., 2012). Ap-1 is also a key transcription factor that induces the apoptosis of endothelial cells and cardiomyocytes (Taimor et al., 2001; Li et al., 2021). Furthermore, AP-1 is a crucial player in the activation of macrophage-induced inflammatory responses (Hannemann et al., 2017). Many drugs inhibit AP-1 expression and thus improve atherosclerosis and ventricular remodeling after acute myocardial infarction (Yoshiyama et al., 2001; Lin et al., 2015; Chou et al., 2019; Yang et al., 2020). PTGS2, a downstream molecule of AP-1, is a key enzyme in AA metabolism. PTGS2 metabolizes AA to produce prostaglandin and thromboxane, which influence platelet aggregation, vascular wall tension, and atherosclerosis. So far, the role of PTGS2 in cardiovascular diseases remains controversial. PTGS2 is generally accepted to promote atherosclerosis; however, it also protects against myocardial ischemia/reperfusion injury (Zhou et al., 2021; Zhuang et al., 2022). CCL2, or monocyte chemoattractant protein-1 (MCP-1), is a well-known CC chemokine that regulates the locomotion and recruitment of monocytes/macrophages to the injury site (Khambhati et al., 2018). CCL2/CCR2 is a key player in the formation, development, and instability of atherosclerotic plaques and has a crucial role in the reconstruction after myocardial infarcts (Dewald et al., 2005; Hernández-Aguilera et al., 2020). Moreover, plasma CCL2 levels have a prognostic value in the acute phase of ACS, with lower CCL2 levels possibly associated with a worse prognosis (Leocádio et al., 2019). Interleukin-8 (CXCL8) is a typical inflammatory mediator, mainly secreted by macrophages. CXCL8 binds to CXCR1 and CXCR2 to perform its role, which can chemotaxis neutrophils to regulate the inflammatory response (Roebuck, 1999). High plasma CXCL8 levels are associated with large infarct size, impaired recovery of left ventricular function, and adverse clinical outcomes (Orn et al., 2009). Furthermore, the CXCL8-induced neutrophil extracellular attractor increases atherosclerosis by activating NF-κB signaling and MAPK in macrophages (An et al., 2019). CSF1 (M-CSF) is a protein that regulates monocyte survival, differentiation, and function (Popova et al., 2011) and is produced in tissues and secreted into the blood. In blood, it recruits monocytes, which then differentiate into tissue macrophages (Roth and Stanley, 1992). Studies have shown that CSF1 deficiency decreases the number of monocytes in peripheral blood and tissues, enhances macrophage apoptosis, and significantly inhibits atherosclerosis (Shaposhnik et al., 2010).

In our study, both high-throughput sequencing and the ELISA test revealed that the JNK1, FOS, AP-1, CCL2, and CXCL8 levels in patients with PBS syndrome were significantly lower than those in healthy adults. This suggests that the JNK/AP-1 signaling pathway might be inhibited in these patients. This could be due to the medications that the patients were taking, such as aspirin and statin, which reduce these inflammatory markers. On the other hand, we are more inclined to conclude that FOS, AP-1, CCL2, CXCL8, and JNK1 were all downregulated by PBS syndrome. We speculate that there are different CHD subtypes, and patients with PBS syndrome exhibit a reduction in some inflammatory markers. We will continue to investigate PBS syndrome in animal models and conduct additional validation of syndrome-related biomarkers.On the other hand, despite the opposing changes in the CSF1 expression in the PBS group during the sequencing and validation stages, we selected CSF1 as an important biomarker as we value the outcomes of basic experiments. Omics research is merely a strategy or a method to determine the potential differential molecules and not to obtain final results. Numerous new directions have opened up through omics research, albeit its findings must be supported by fundamental studies. In our study, the sample size for ELISA verification was much larger than that for omics sequencing, which provided greater confidence in the ELISA results. In other words, we focused more on the implementation of clinical validation and the omics technology played an auxiliary role. Therefore, we consider the upregulation of CSF1 as the characteristic of PBS syndrome of coronary heart disease. In addition, we did not perform diagnostic tests of omics data of NPBS syndrome, which could have benefited in determining whether the biomarkers of CHD-PBS syndrome are altered in the CHD-NPBS syndrome. These are the limitations of the study. In summary, this study aimed to ascertain the biological foundation of CHD-PBS syndrome rather than the biological basis of the CHD-NPBS syndrome or the distinction between the two syndromes. In the future, we plan to conduct an in-depth analysis to look at the distinct molecules across diverse syndromes.

## Conclusion

By integrating the clinical samples with multi-omics studies, the present study identified that 1) the “gene–protein–metabolite” network of CHD-PBS syndrome includes at least 33 mRNAs, four proteins, and 25 metabolites. 2) The disorder of AA metabolism and the inhibition of the JNK/AP-1 pathway may be the characteristics of PBS syndrome. 3) PBS syndrome exhibited decreased levels of FOS, AP-1, CCL2, CXCL8, and JNK1 and increased levels of PTGS2 and CSF1. 4) The diagnostic biomarkers of CHD-PBS syndrome included CSF1 (>286.6 pg/ml), PTGS2 (>992.7 pg/ml), and CXCL8 (>1479 pg/ml). This study provides a methodological reference for the analyses of the biological foundation of TCM under objective, quantitative, and accurate requirements and suggests that TCM syndromes, which are a subtype of diseases, may have unique molecular changes.

## Data Availability

The RNA-seq data have been deposited to the SRA database with the dataset identifier PRJNA871404. The mass spectrometry mics data have been deposited to the Xchange Consortium (http://partnerrepositorywiththedatasetcentral.proteomexchange.org) *via* XD036389.

## Abbreviations

AA, Arachidonic acid; ACS, Acute coronary syndrome; AUC, Area under the receiver operating curve; AUC, Area under the receiver operating curve; CHD, Coronary heart disease; DDA, Data dependent acquisition; DGs, Differential genes; DMs, Differential metabolites; DPs, Differential proteins; Elisa, Enzyme linked immunosorbent assay; FC, Fold change; HC, Healthy control; KEGG, Kyoto Encyclopedia of Genes and Genomes; LC-MS, Liquid chromatography tandem mass spectrometry; NPBS, Non phlegm and blood stasis syndrome; OPLS-DA, Orthogonal partial least-squares-discriminant analysis; PBS, Phlegm and Blood Stasis; PPI, Protein-protein interaction; ROC, Receiver operating characteristic curve; TCM, traditional Chinese medicine; VIP, Variable importance in the projection.
