# Supplementary material for: Exploring the “gene–protein–metabolite” network of coronary heart disease with phlegm and blood stasis syndrome by integrated multi-omics strategy
Source: Front Pharmacol. 2022 Nov 29;13:1022627. doi: 10.3389/fphar.2022.1022627 (PMC9744761; doi:10.3389/fphar.2022.1022627)
Supplement: Supplementary file 2 [file DataSheet2.docx]

# Fig. S1 Boxplot for FPKM Values in different groups.

#
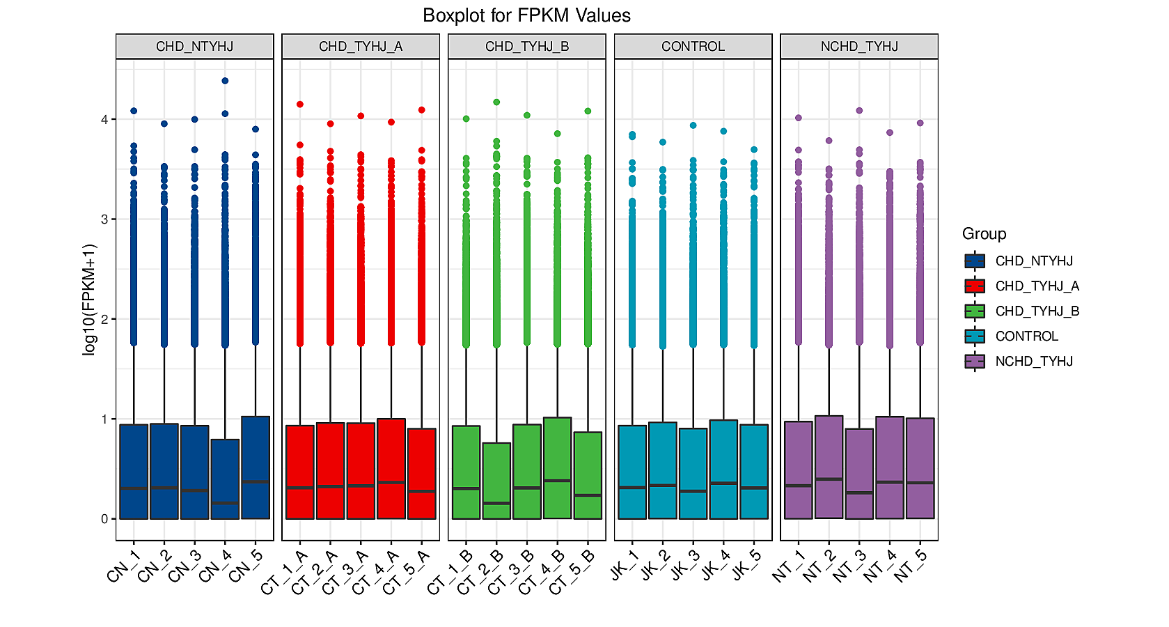


# Fig. S2 KEGG enrichment analysis of differentially expressed genes in different groups.

#
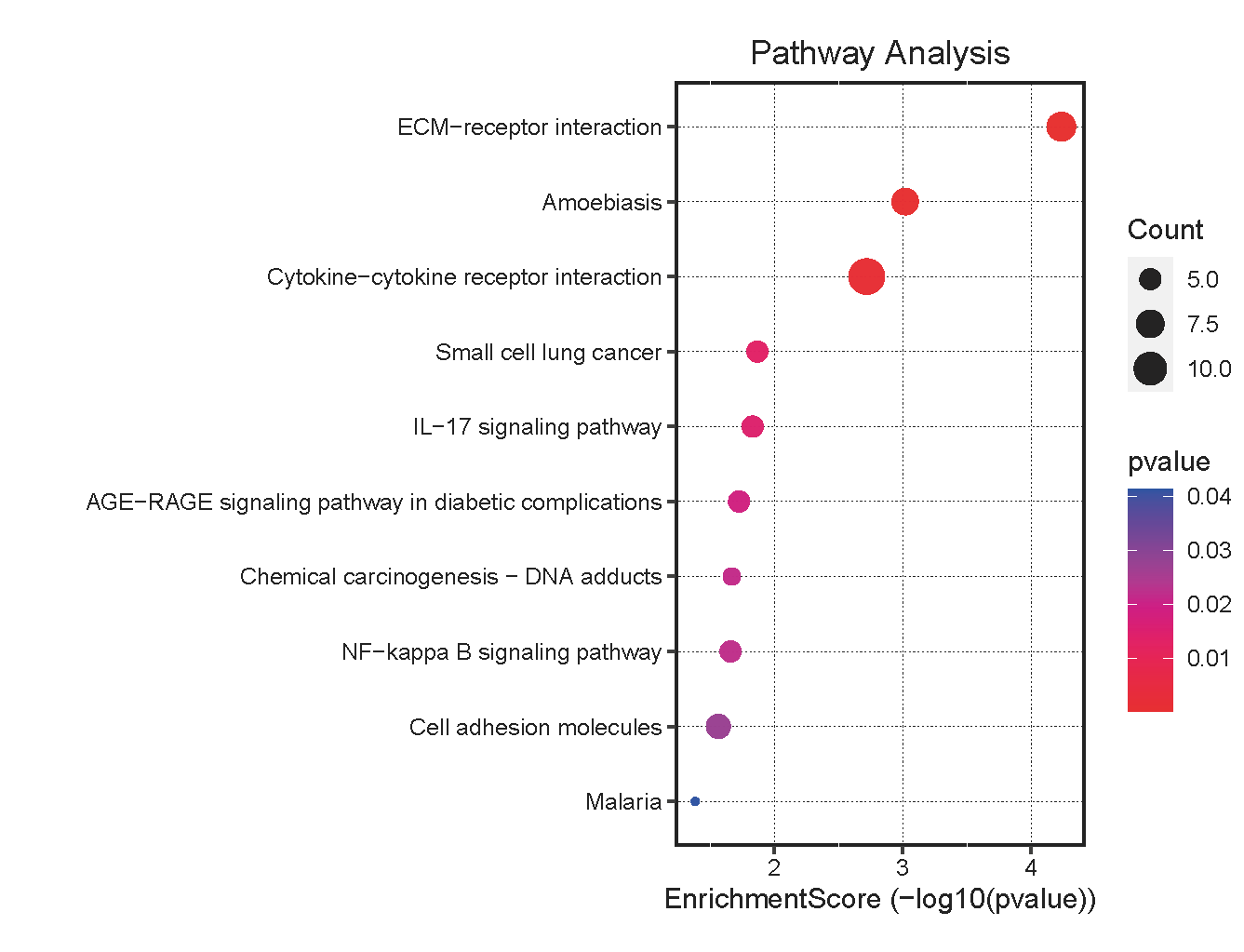

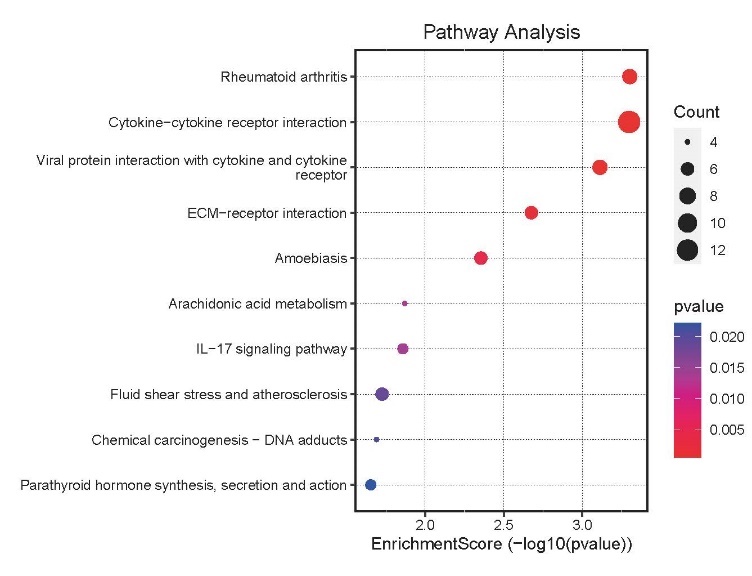

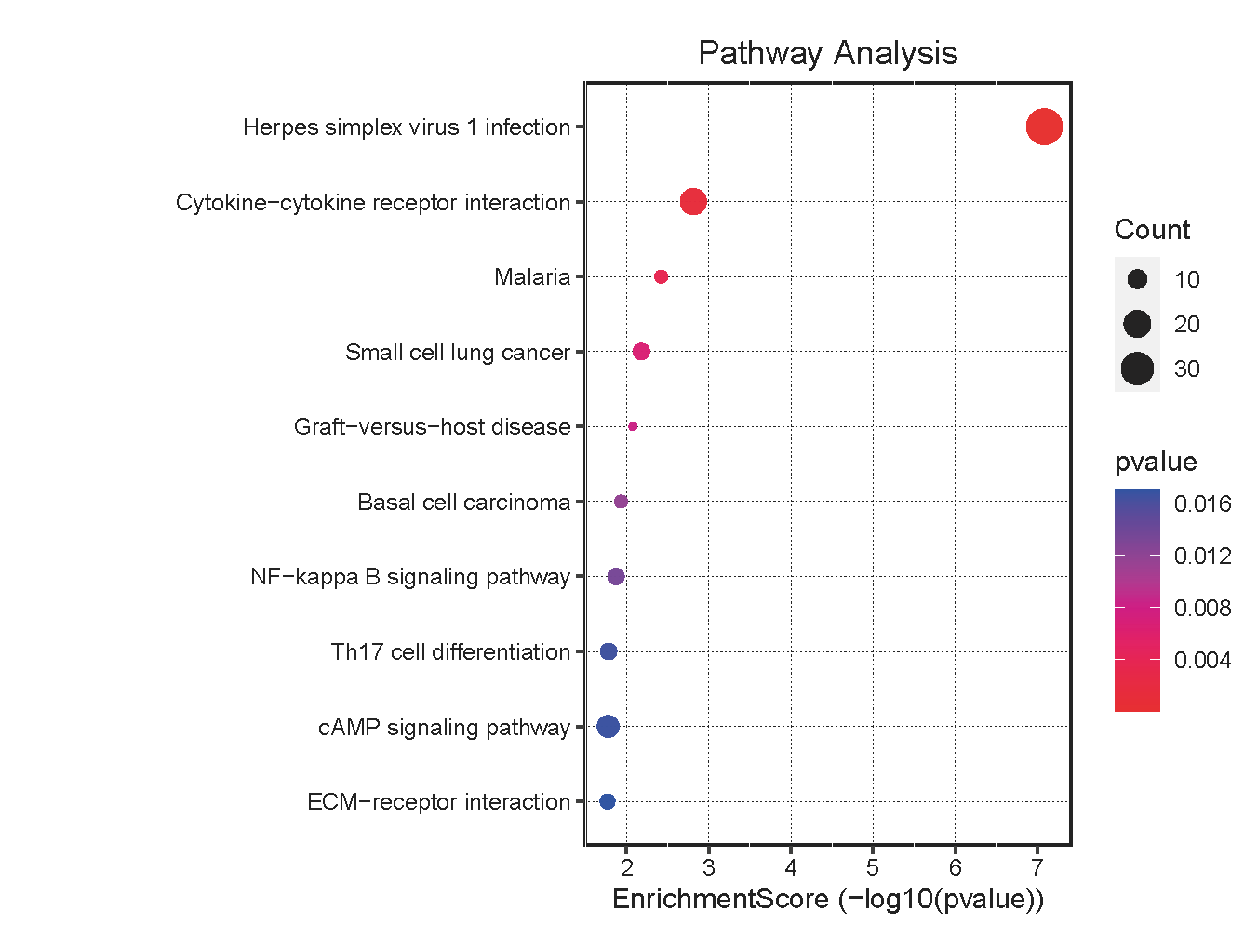
Fig. S3 Deviation of m/z with & without calibration.

B

C

A


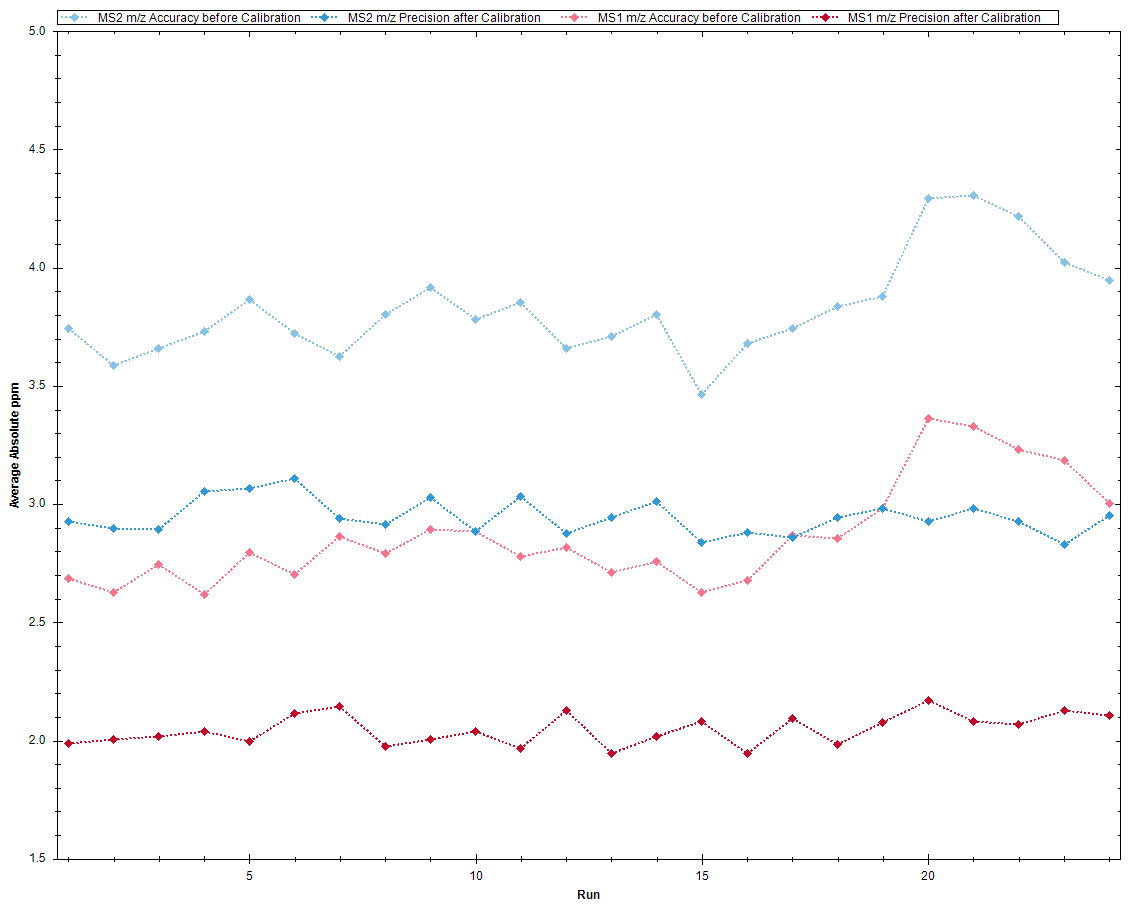


# Fig. S4 Data points per peak.


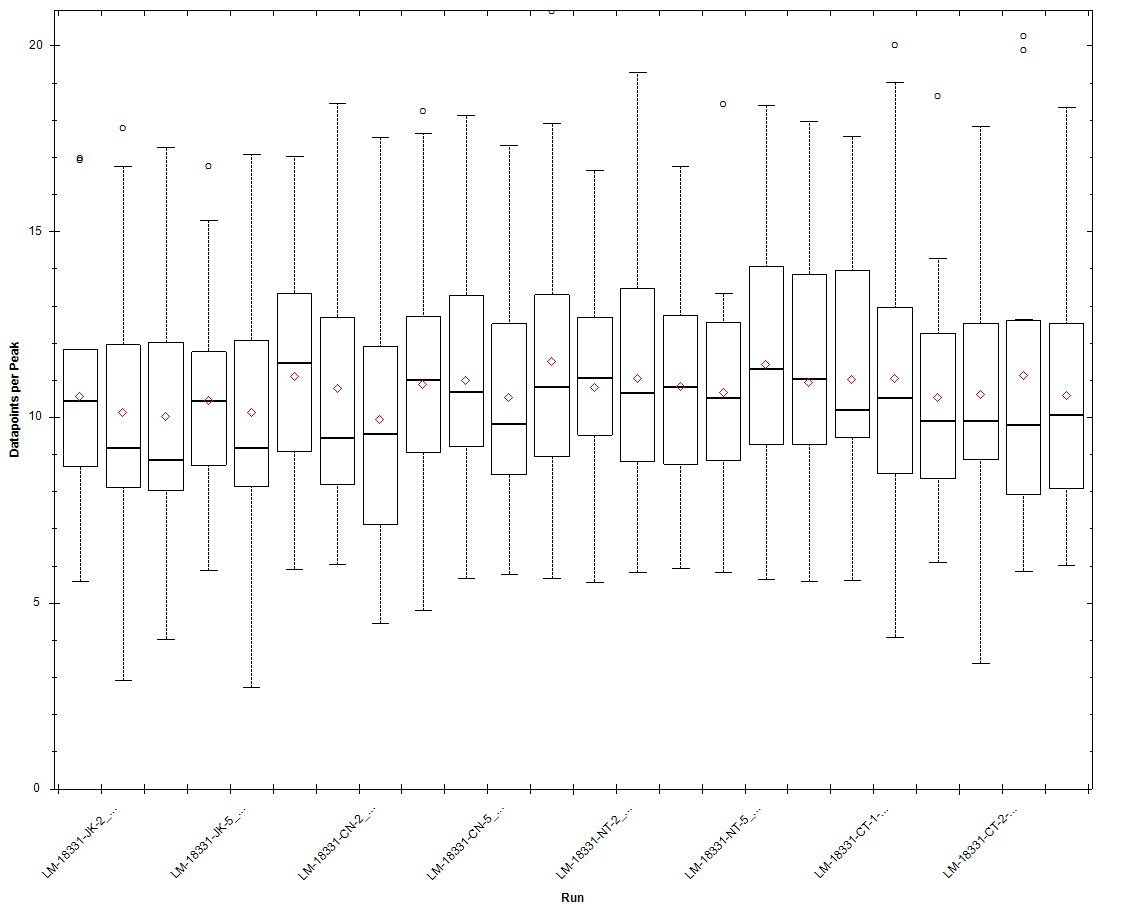


# Fig. S5 Correlation analysis of MIX-QC quantitative data.


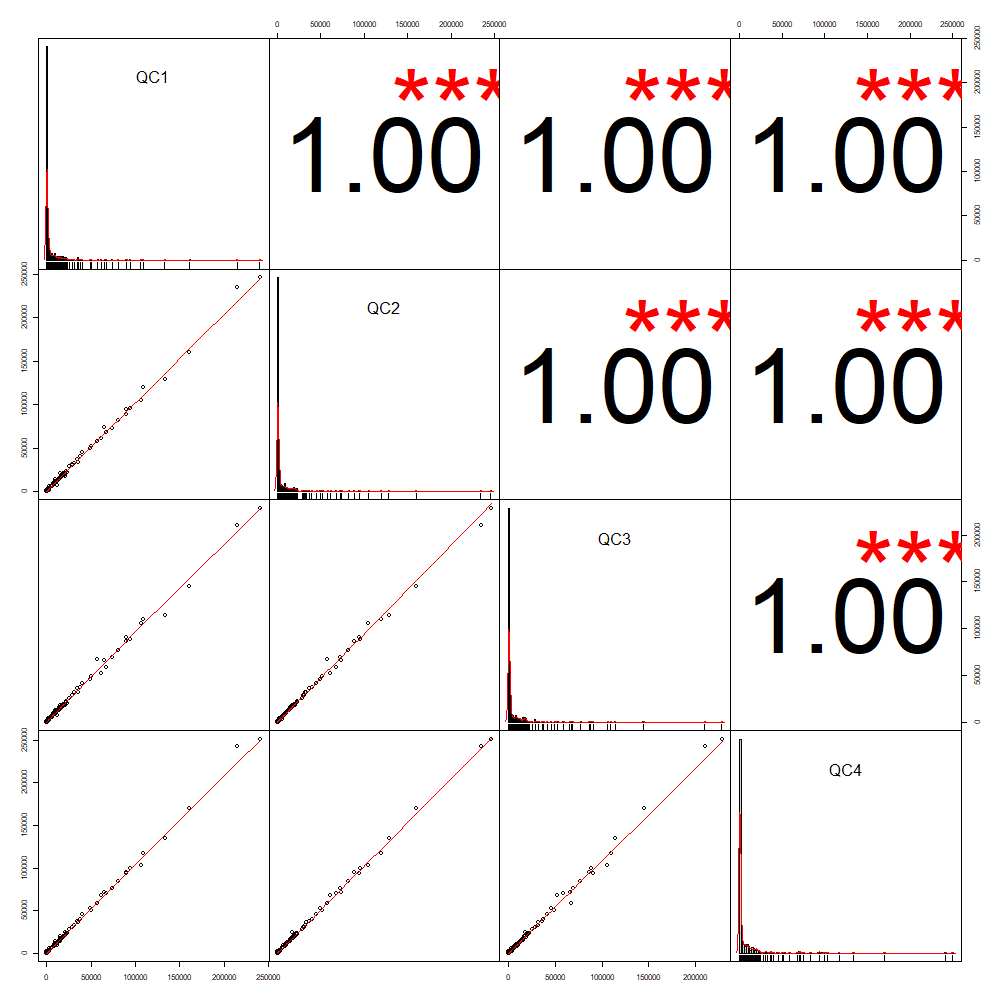


**
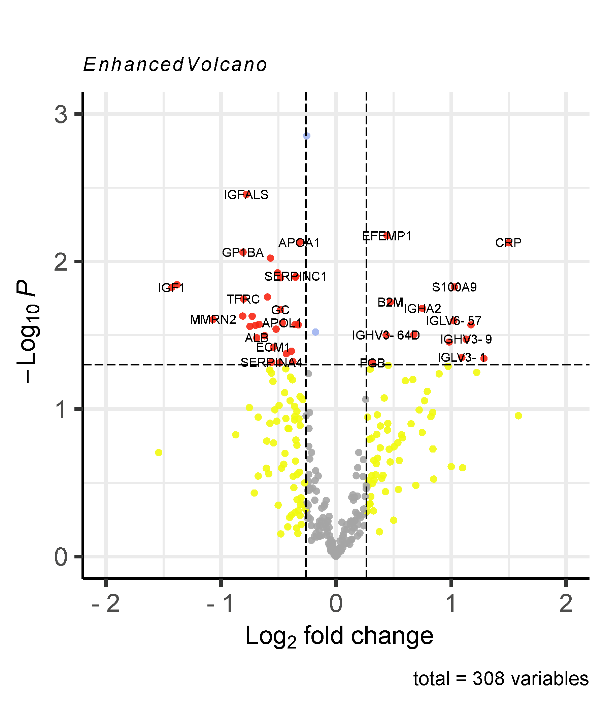
Fig**. **S6 The volcano diagrams of the differential proteins for PBS and NPBS Syndrome.**

**
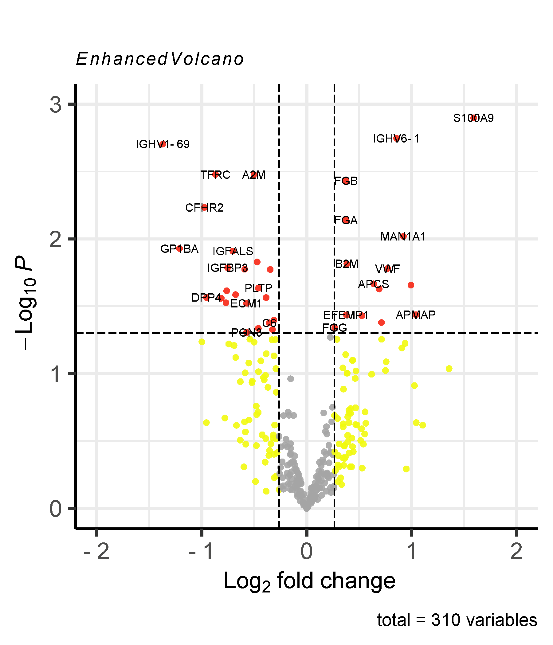
**

**Fig**. **S7** A Base Peak Chromatogram for positive and negative ion.


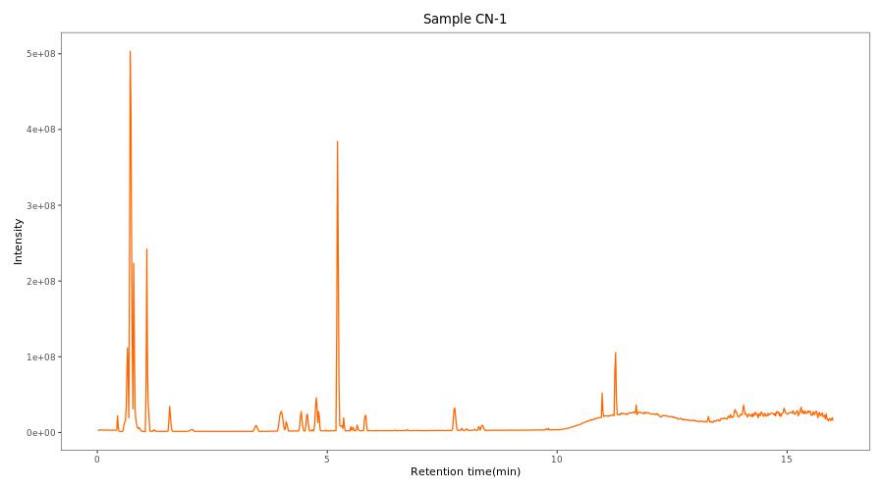

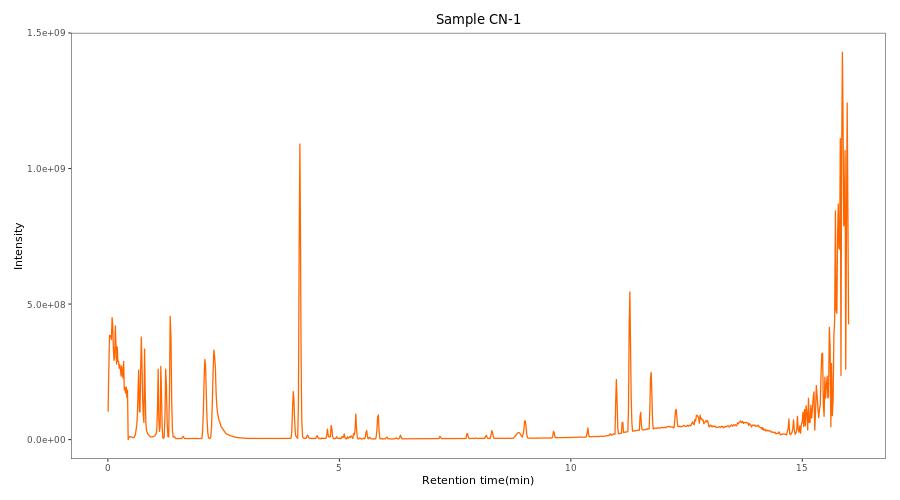


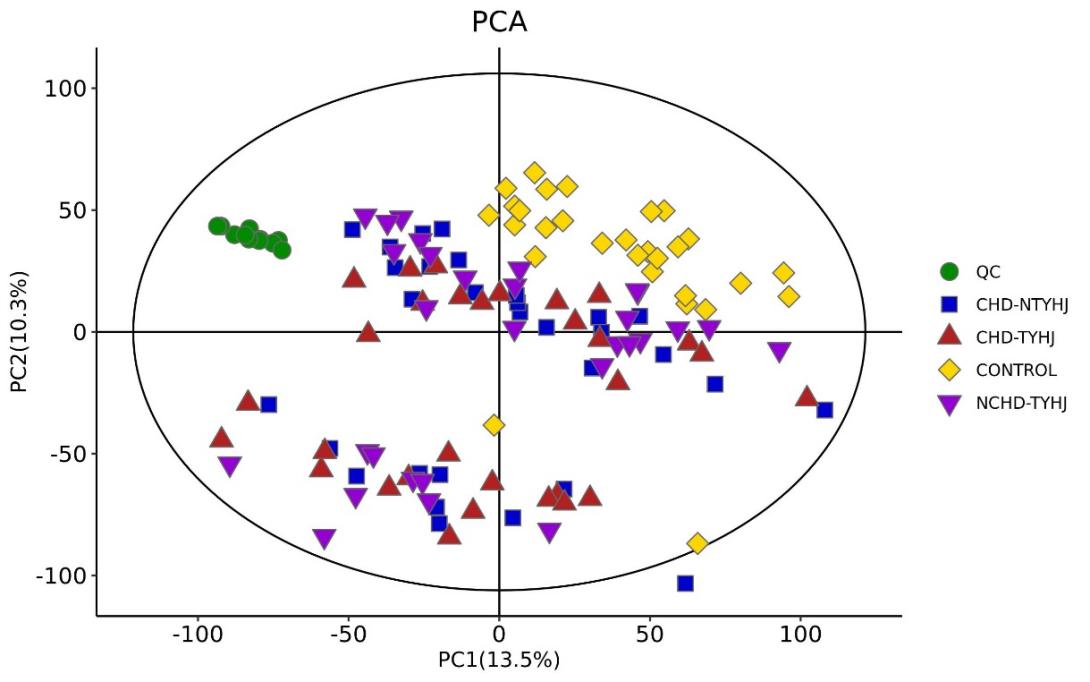
**Fig**. **S8** Principal component analysis of QC samples.

**Fig**. **S9** Boxplot of metabolite intensities of QC samples
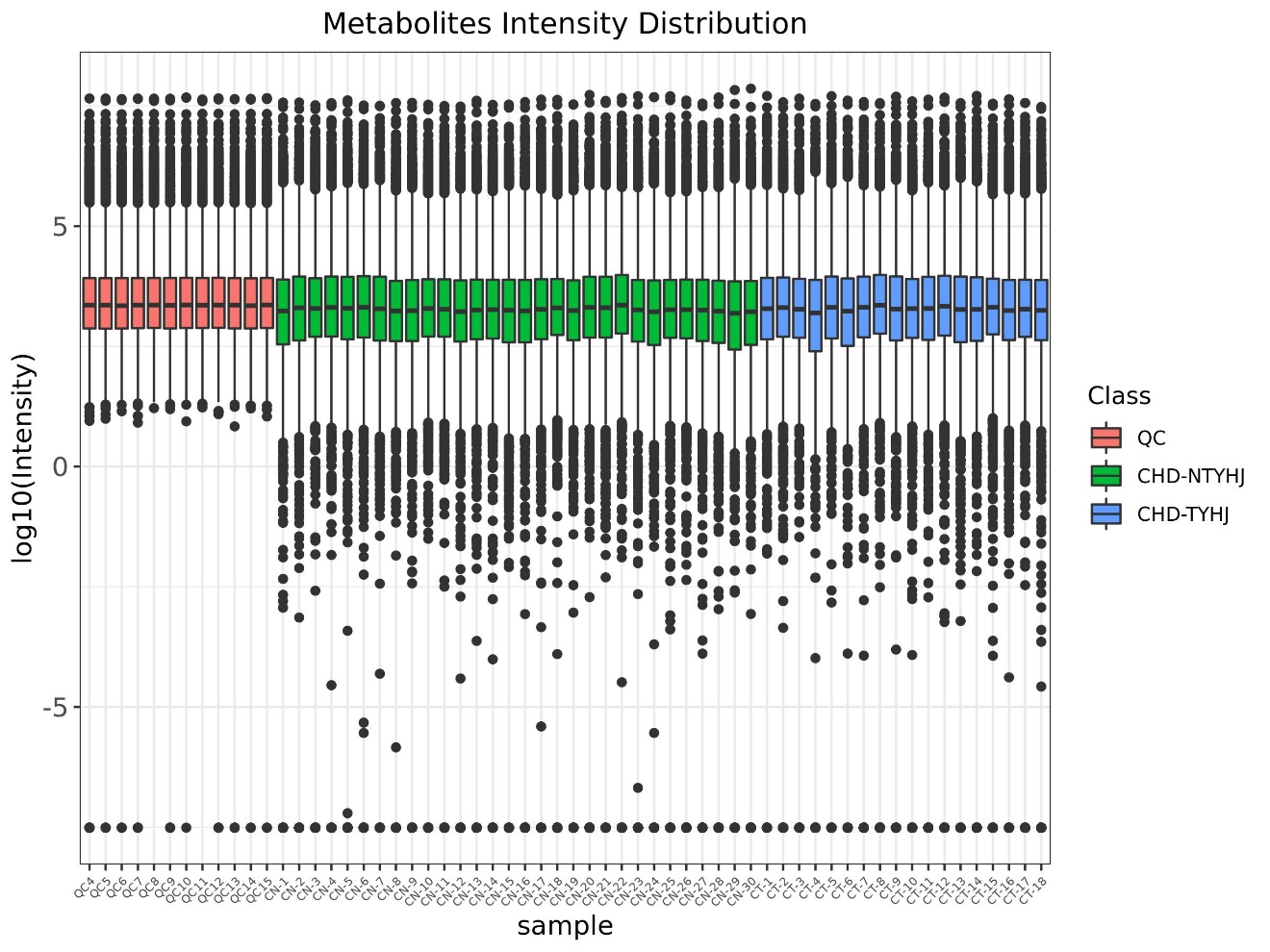
.
