# Supplementary material for: Exploring the “gene–protein–metabolite” network of coronary heart disease with phlegm and blood stasis syndrome by integrated multi-omics strategy
Source: Front Pharmacol. 2022 Nov 29;13:1022627. doi: 10.3389/fphar.2022.1022627 (PMC9744761; doi:10.3389/fphar.2022.1022627)
Supplement: Supplementary file 3 [file DataSheet1.docx]

**Table. S1 Diagnostic criteria for PBS Syndrome.**

| Syndrome diagnosis | Symptom | Score |
| --- | --- | --- |
| Blood stasis Syndrome | fixed chest pain | 4 |
|  | Tongue is dark or ecchymosis | 4 |
|  | Sublingual vein is dark or purple | 3 |
|  | purple and dark complexion | 3 |
|  | body has ecchymosis or spots | 3 |
|  | limb numbness | 2 |
|  | dark and purple tongue | 2 |
|  | Uneven pulse | 2 |
| Phlegm Syndrome | chest distress | 3 |
|  | excessive phlegm and fat | 3 |
|  | Thick and greasy tongue coating | 3 |
|  | The stool is sticky and greasy | 2 |
|  | Heavy limbs | 2 |
|  | dizzy and sleepy | 2 |
|  | Sticky mouth | 2 |
|  | slippery Pulse | 2 |

The individual Syndrome is diagnosed if the sum of scores is > 8.

**Table. S2 Characteristics of the participants in RNA-seq.**

| Participants | CHD PBS | CHD NPBS | HC |
| --- | --- | --- | --- |
|  | （n=5） | （n=5） | （n=5） |
| Age | 60.20±13.63 | 63.20±3.49 | 25±1.87 |
| Sex（male%） | 4（80.00%） | 3（60.00%） | 3（60.00%） |
| Hypertension（%） | 4（80.00%） | 3（60.00%） | 0 |
| Hyperlipidemia（%） | 2（40.00%） | 5（100.00%） | 0 |
| Diabetes（%） | 3（60.00%） | 1（20.00%） | 0 |

**Table. S3 Characteristics of the participants in DIA-based proteomics .**

| Participants | CHD PBS | CHD NPBS | HC |
| --- | --- | --- | --- |
|  | （n=6） | （n=6） | （n=6） |
| Age | 59.33±12.37 | 63.17±3.13 | 25.00±1.67 |
| Sex（male%） | 5（83.33%） | 4（66.67%） | 3（50.00%） |
| Hypertension（%） | 4（66.67%） | 3（50.00%） | 0 |
| Hyperlipidemia（%） | 3（50.00%） | 5（83.33%） | 0 |
| Diabetes（%） | 3（50.00%） | 2（33.33%） | 0 |

**Table. S4 Characteristics of the participants in metabolomics.**

| Participants | CHD PBS | CHD NPBS | HC |
| --- | --- | --- | --- |
|  | （n=30） | （n=30） | （n=30） |
| Age | 60.04±7.40 | 65.37±3.91 | 25.03±1.92 |
| Sex（male%） | 23（76.67%） | 15（50.00%） | 16（53.33%） |
| Hypertension（%） | 27（90.00%） | 20（66.67%） | 0 |
| Hyperlipidemia（%） | 22（73.33%） | 27（90.00%） | 0 |
| Diabetes（%） | 12（40.00%） | 11（36.67%） | 0 |

**Table. S5 Characteristics of the participants in ELISA validation.**

| Participants | CHD PBS | HC |
| --- | --- | --- |
|  | （n=64） | （n=30） |
| Age | 60.99±7.10 | 57.24±5.51 |
| Sex（male%） | 41（64.06 %） | 17（53.33%） |
| Hypertension（%） | 39（60.94%） | 0 |
| Hyperlipidemia（%） | 51（79.69%） | 0 |
| Diabetes（%） | 18（28.13%） | 0 |

# Table. S6 The quality assessment for the raw data of RNA-seq.

| Sample | RawReads | RawBases | CleanReads | CleanBases | ValidBases | Q30 | GC |
| --- | --- | --- | --- | --- | --- | --- | --- |
| NPBS_1 | 48.55M | 7.28G | 48.17M | 6.94G | 0.9526 | 0.9501 | 0.4937 |
| NPBS_2 | 50.57M | 7.59G | 50.17M | 7.23G | 0.9526 | 0.9488 | 0.488 |
| NPBS_3 | 47.11M | 7.07G | 46.75M | 6.67G | 0.944 | 0.9513 | 0.4905 |
| NPBS_4 | 49.04M | 7.36G | 48.65M | 6.98G | 0.9493 | 0.9486 | 0.5005 |
| NPBS_5 | 48.40M | 7.26G | 48.02M | 6.87G | 0.9466 | 0.9505 | 0.4971 |
| HC_1 | 48.26M | 7.24G | 47.90M | 6.90G | 0.9534 | 0.9525 | 0.4877 |
| HC_2 | 49.99M | 7.50G | 49.59M | 7.10G | 0.9474 | 0.953 | 0.4894 |
| HC_3 | 50.69M | 7.60G | 50.32M | 7.23G | 0.9506 | 0.9554 | 0.491 |
| HC_4 | 48.65M | 7.30G | 48.31M | 6.91G | 0.9472 | 0.9537 | 0.4898 |
| HC_5 | 51.16M | 7.67G | 50.65M | 7.37G | 0.96 | 0.9345 | 0.4918 |
| PBS_1 | 49.22M | 7.38G | 48.83M | 7.02G | 0.9509 | 0.9552 | 0.4941 |
| PBS_2 | 51.05M | 7.66G | 50.64M | 7.34G | 0.958 | 0.9495 | 0.4886 |
| PBS_3 | 49.66M | 7.45G | 49.27M | 7.05G | 0.9469 | 0.9509 | 0.4956 |
| PBS_4 | 47.10M | 7.06G | 46.72M | 6.77G | 0.9576 | 0.9489 | 0.4984 |
| PBS_5 | 49.00M | 7.35G | 48.63M | 7.00G | 0.9527 | 0.9546 | 0.4913 |

# Table. S7 The results of the reference genome comparison

| Sample | Total reads | Total mapped reads | Multiple mapped | Read-1 | Read-2 | Reads map to '+' | Reads map to '-' | Splice reads | Reads mapped in proper pairs |
| --- | --- | --- | --- | --- | --- | --- | --- | --- | --- |
| NPBS_1 | 48165722 | 47192953(97.98%) | 1622844(3.37%) | 22787857(47.31%) | 22782252(47.30%) | 22775750(47.29%) | 22794359(47.32%) | 14055409(29.18%) | 44287528(91.95%) |
| NPBS_2 | 50168456 | 49089760(97.85%) | 1507482(3.00%) | 23787780(47.42%) | 23794498(47.43%) | 23779342(47.40%) | 23802936(47.45%) | 13664578(27.24%) | 46212936(92.12%) |
| NPBS_3 | 46748494 | 45664236(97.68%) | 1432527(3.06%) | 22117242(47.31%) | 22114467(47.31%) | 22108664(47.29%) | 22123045(47.32%) | 11765117(25.17%) | 42976058(91.93%) |
| NPBS_4 | 48651122 | 47686386(98.02%) | 2344765(4.82%) | 22673892(46.61%) | 22667729(46.59%) | 22659700(46.58%) | 22681921(46.62%) | 13715594(28.19%) | 44065870(90.58%) |
| NPBS_5 | 48018660 | 46965232(97.81%) | 1679145(3.50%) | 22644652(47.16%) | 22641435(47.15%) | 22633306(47.13%) | 22652781(47.17%) | 14960510(31.16%) | 43952516(91.53%) |
| PBS_1 | 48829194 | 47746038(97.78%) | 1659268(3.40%) | 23033492(47.17%) | 23053278(47.21%) | 23037644(47.18%) | 23049126(47.20%) | 14202220(29.09%) | 44836998(91.82%) |
| PBS_2 | 50640710 | 49745863(98.23%) | 1553604(3.07%) | 24099779(47.59%) | 24092480(47.58%) | 24086474(47.56%) | 24105785(47.60%) | 15331753(30.28%) | 46975712(92.76%) |
| PBS_3 | 49266372 | 48257954(97.95%) | 1589196(3.23%) | 23340352(47.38%) | 23328406(47.35%) | 23325900(47.35%) | 23342858(47.38%) | 14539574(29.51%) | 45324622(92.00%) |
| PBS_4 | 46721712 | 45913476(98.27%) | 1433549(3.07%) | 22247049(47.62%) | 22232878(47.59%) | 22232291(47.58%) | 22247636(47.62%) | 13905902(29.76%) | 43343276(92.77%) |
| PBS_5 | 48632708 | 47624253(97.93%) | 1601012(3.29%) | 23011394(47.32%) | 23011847(47.32%) | 23002208(47.30%) | 23021033(47.34%) | 14379803(29.57%) | 44677602(91.87%) |
| HC_1 | 47896368 | 46869159(97.86%) | 1484480(3.10%) | 22690935(47.38%) | 22693744(47.38%) | 22684653(47.36%) | 22700026(47.39%) | 13048300(27.24%) | 44112456(92.10%) |
| HC_2 | 49591906 | 48496169(97.79%) | 1505951(3.04%) | 23489039(47.36%) | 23501179(47.39%) | 23484811(47.36%) | 23505407(47.40%) | 13451569(27.12%) | 45670276(92.09%) |
| HC_3 | 50316058 | 49254300(97.89%) | 1608149(3.20%) | 23821405(47.34%) | 23824746(47.35%) | 23814495(47.33%) | 23831656(47.36%) | 13786544(27.40%) | 46302662(92.02%) |
| HC_4 | 48311740 | 47364321(98.04%) | 1526636(3.16%) | 22918689(47.44%) | 22918996(47.44%) | 22911174(47.42%) | 22926511(47.46%) | 13819120(28.60%) | 44498662(92.11%) |
| HC_5 | 50651842 | 49482238(97.69%) | 1481005(2.92%) | 24000189(47.38%) | 24001044(47.38%) | 23991163(47.36%) | 24010070(47.40%) | 14142324(27.92%) | 46821500(92.44%) |

**Table. S8 Comparison of FPKM homogeneity**

| sample | Min | 1st_Qu | Median | Mean | 3rd_Qu | Max | Sd | Sum |
| --- | --- | --- | --- | --- | --- | --- | --- | --- |
| NPBS_1 | 0.0 | 0.0 | 1.0 | 17.7 | 7.7 | 12107.1 | 136.1 | 354333.6 |
| NPBS_2 | 0.0 | 0.0 | 1.0 | 16.0 | 7.9 | 9006.2 | 108.1 | 320604.2 |
| NPBS_3 | 0.0 | 0.0 | 0.9 | 16.3 | 7.5 | 9941.4 | 116.9 | 326802.4 |
| NPBS_4 | 0.0 | 0.0 | 0.4 | 16.5 | 5.2 | 24224.5 | 213.9 | 329620.7 |
| NPBS_5 | 0.0 | 0.0 | 1.3 | 19.4 | 9.5 | 7936.5 | 128.3 | 388494.3 |
| PBS_1 | 0.0 | 0.0 | 1.0 | 17.4 | 7.5 | 14093.5 | 143.2 | 348687.0 |
| PBS_2 | 0.0 | 0.0 | 1.0 | 15.9 | 7.5 | 10093.3 | 112.6 | 318185.8 |
| PBS_3 | 0.0 | 0.0 | 1.1 | 17.5 | 8.1 | 9001.3 | 119.5 | 349651.4 |
| PBS_4 | 0.0 | 0.0 | 0.4 | 17.2 | 4.7 | 14831.0 | 162.9 | 344320.2 |
| PBS_5 | 0.0 | 0.0 | 1.1 | 17.1 | 8.0 | 10764.8 | 125.3 | 341586.9 |
| HC_1 | 0.0 | 0.0 | 1.1 | 16.1 | 7.5 | 7042.4 | 109.2 | 321698.0 |
| HC_2 | 0.0 | 0.0 | 1.2 | 15.6 | 8.2 | 5891.6 | 90.8 | 313040.8 |
| HC_3 | 0.0 | 0.0 | 0.9 | 15.8 | 7.0 | 8668.5 | 108.0 | 316992.1 |
| HC_4 | 0.0 | 0.0 | 1.3 | 16.5 | 8.7 | 7579.3 | 103.8 | 330889.4 |
| HC_5 | 0.0 | 0.0 | 1.0 | 15.3 | 7.7 | 4966.1 | 91.5 | 306459.6 |
